# Inhalable Formulation Based on Lipid–Polymer Hybrid Nanoparticles for the Macrophage Targeted Delivery of Roflumilast

**DOI:** 10.1021/acs.biomac.2c00576

**Published:** 2022-07-28

**Authors:** Emanuela F. Craparo, Marta Cabibbo, Cinzia Scialabba, Gaetano Giammona, Gennara Cavallaro

**Affiliations:** † Lab of Biocompatible Polymers, Department of Biological, Chemical and Pharmaceutical Sciences and Technologies (STEBICEF), University of Palermo, Via Archirafi 32, Palermo90123, Italy; ‡ Consorzio Interuniversitario Nazionale per la Scienza e Tecnologia dei Materiali (INSTM) of Palermo, Palermo, Italy; § Advanced Technology and Network Center (ATeN Center), Università di Palermo, Palermo90133, Italy

## Abstract

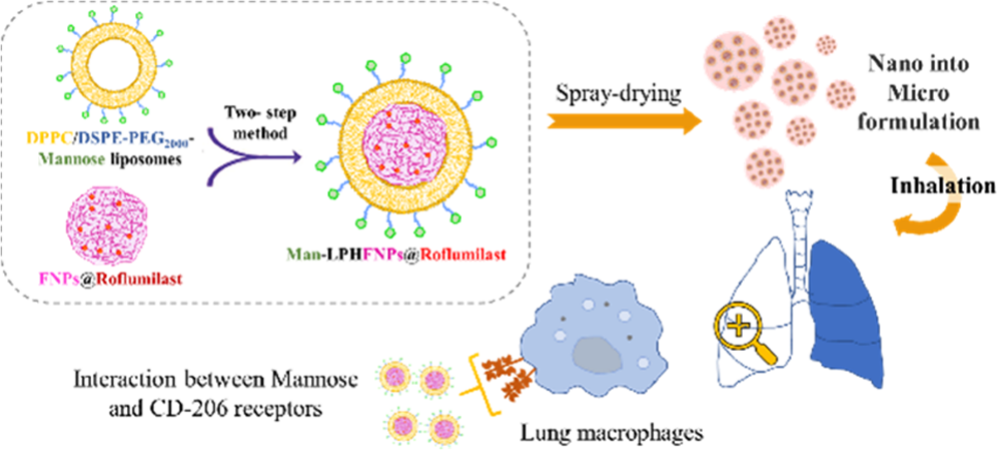

Here, novel lipid–polymer hybrid nanoparticles (LPHNPs), targeted to lung macrophages, were realized as potential carriers for Roflumilast administration in the management of chronic obstructive pulmonary disease (COPD). To achieve this, Roflumilast-loaded fluorescent polymeric nanoparticles, based on a polyaspartamide-polycaprolactone graft copolymer, and lipid vesicles, made from 1,2-dipalmitoyl-*sn*-glycero-3-phosphocholine and 1,2-distearoyl-*sn*-glycero-phosphoethanolamine-*N*-(polyethylene glycol)-mannose, were properly combined using a two-step method, successfully obtaining Roflumilast-loaded hybrid fluorescent nanoparticles (Man-LPHFNPs@Roflumilast). These exhibit colloidal size and a negative ζ potential, 50 wt % phospholipids, and a core–shell-type morphology; they slowly release the entrapped drug in a simulated physiological fluid. The surface analysis also demonstrated their high surface PEG density, which confers mucus-penetrating properties. Man-LPHFNPs@Roflumilast show high cytocompatibility toward human bronchial epithelium cells and macrophages and are uptaken by the latter through an active mannose-mediated targeting process. To achieve an inhalable formulation, the nano-into-micro strategy was applied, encapsulating Man-LPHFNPs@Roflumilast in poly(vinyl alcohol)/leucine-based microparticles by spray-drying.

## Introduction

Nanomedicine approaches have incredible potential for managing many serious diseases due to the ability of smart nanostructured systems to optimize bioavailability and enable targeted delivery of various therapeutic or diagnostic agents.^1^ The most promising nanomedicine is based on polymeric nanoparticles or liposomes, some of which were already approved by the US FDA for clinical use, thanks to versatile drug loading and controlled release, high cellular uptake, storage and biological stability of nanoparticles, and excellent biocompatibility and long circulation half-life of liposomes.^1,2^

Recently, researchers have sought to combine their advantages to overcome some of their limitations, especially related to liposomes, such as structural disintegration and drug leakage, by designing lipid–polymer hybrid nanoparticles (LPHNPs).^2^ Structurally, LPHNPs have a core–shell structure consisting of a polymeric core coated by a phospholipid layer, which provides a highly biocompatible shell and increases drug retention inside the polymeric core. Lipid–PEG derivates may be embedded either as a steric stabilizer or as a linker for conjugation of targeting ligands. This unique structural design guarantees high structural integrity and biocompatibility, the ability to load multiple therapeutic and imaging agents, storage stability, molecular targeting properties, and well-defined release kinetics.^2^ Moreover, LPHNPs can be administered by all possible routes, also the pulmonary one, optimizing the bioavailability and reducing the undesirable effects of some drugs compared to when they are administered by conventional dosage forms.^3^

Phosphodiesterases-4 (PDE4) are a superfamily of enzymes (PDE4A-D), whose levels are significantly augmented in many inflammatory and immune lung cells, involved in the pathogenesis of chronic inflammatory diseases, such as asthma and chronic obstructive pulmonary disease (COPD).^4^ Alveolar macrophages (AMs) play a key role in the pathophysiology of COPD, as they are activated by cigarette smoke and other irritants to release inflammatory mediators.^5^ Due to the overexpression of PDE4 in AMs implicated in COPD, the use of PDE4 inhibitors could be a valid anti-inflammatory strategy able to inhibit macrophage functions in COPD.^4^

Roflumilast (3-cyclo-propylmethoxy-4-difluoromethoxy-*N*-[3,5-dichloropyrid-4-yl]-benzamide) is the only PDE4 inhibitor approved by the US FDA and the European Medicines Agency (EMA) as an oral, once-daily tablet for the treatment of severe COPD associated with chronic bronchitis.^4^ In particular, it improves airway remodeling, ventilation, and mucociliary functions, reduces oxygen free radical release, inhibits pulmonary fibrosis, and shows anti-inflammatory effects. Despite these benefits, oral Roflumilast comes with severe psychiatric and gastrointestinal side effects, which limit its clinical use.^4,6^ Therefore, the inhalation route could represent a potential approach to improve the therapeutic index of Roflumilast, being delivered directly to the site of action, with smaller doses, reducing gastrointestinal and systemic exposure.^7,8^ It was recently reported in the literature that inhaled Roflumilast was more effective than that orally administered in curbing and relieving allergen-induced airflow obstructions in Brownian Norway rats.^9^

Considering the need to improve the lung bioavailability of Roflumilast for the treatment of COPD, and the therapeutic potential of LPHNPs, in this paper, we have developed an innovative inhalable formulation for Roflumilast based on LPHNPs to achieve a targeted drug release to the AMs, the main cells that orchestrate the inflammatory response in the COPD.

These carriers were realized by combining two components: a polymeric core, containing Roflumilast, made of a biocompatible polycaprolactone (PCL)/ α,β-poly(*N*-2-hydroxyethyl)-dl-aspartamide (PHEA) graft copolymer; and a lipid shell, obtained by a mixture between 1,2-dipalmitoyl-*sn*-glycero-3-phosphocholine (DPPC) and 1,2-distearoyl-*sn*-glycero-3-phosphoethanolamine-*N*-(polyethylene glycol)_2000_ conjugated to mannose (DSPE-PEG_2000_-mannose), which is a CD-206-targeted ligand, overexpressed on AMs of COPD patients.^10,11^ LPHNPs were produced by high-pressure homogenization (HPH) and characterized in terms of phospholipid and Roflumilast content, mean size, morphology, surface properties, thermal behavior, drug release profile, and interactions with mucin. Preliminary in vitro studies were carried out to evaluate the absence of cell toxicity and determine the targeting function of mannose toward the macrophages. To achieve an inhalable formulation, the nano-into-micro (NiM) strategy was followed.^12,13^ In particular, an inhalable powder consisting of microparticles based on poly(vinyl alcohol) (PVA) and l-leucine (Leu), where LPHNPs were homogenously dispersed, was obtained by spray-drying.^14^

## Materials and Methods

### Materials

Anhydrous *N*,*N*′-dimethylformamide (a-DMF), poly-ε-caprolactone (PCL, *M̅*_w_ = 10,000–18,000 Da), Rhodamine B (RhB), succinic anhydride (SA), 4-dimethylaminopyridine (4-DMAP), 1,10-carbonyldiimidazole (CDI), poly(vinyl alcohol) (*M̅*_w_ = 31,000–50,000) (PVA), l-leucine (Leu), 1,2-dipalmitoyl-*sn*-glycero-3-phosphocholine (DPPC), diethyl ether, dichloromethane, ethanol, chloroform, acetonitrile (ACN), (poly(ethylene glycols) standards, dimethylacetamide (DMA), iron (III) chloride hexahydrate, ammonium thiocyanate, Dulbecco’s phosphate-buffered saline (DPBS), diethylenetriaminepentaacetic acid (DTPA), Roswell Park Memorial Institute (RPMI) amino acid solution, type II mucine from porcine stomach, and egg yolk emulsion were purchased from Sigma-Aldrich (Milan, Italy). Diethylamine (DEA) and sodium chloride (NaCl) were obtained from Fluka (Milan, Italy). Hydroxyethyl cellulose (HEC) and potassium chloride (KCl) were purchased from Carlo Erba. 1,2-Distearoyl-*sn*-glycero-3-phosphoethanolamine-*N*-(polyethylene glycol)_2000_-mannose (DSPE-PEG_2000_-mannose) was purchased from Ruixibiotech (Xi’an, China). Roflumilast was obtained from abcr GmbH (Karlsruhe, Germany). Dialysis membranes were purchased from Spectrum Labs.

α,β-Poly(*N*-2-hydroxyethyl)-dl-aspartamide (PHEA) and PHEA-g-RhB were prepared and purified according to a previously reported procedure.^15^

### Synthesis and Characterization of Poly-ε-caprolactone-succinate (PCL-Succ)

Carboxyl-terminated PCL was synthesized according to a recently reported procedure.^16^ Briefly, to a solution of PCL in a-DMF (133.4 mg/mL) were added 4-DMAP (*R*_2_ = mmol DMAP/mmol PCL = 1.2) and SA (*R*_1_ = mmol SA/mmol PCL = 40). After stirring for 24 h at 60 °C, the reaction mixture was precipitated in cold diethyl ether. The obtained precipitate was washed four times with twice-distilled water. Finally, the solid product was dissolved in acetone, dialyzed against twice-distilled water (MWCO 3.5 kDa), and freeze-dried using a freeze-dryer.

^1^H-NMR spectra were registered by a Bruker Advance II-300 spectrometer, working at 300 MHz (Bruker, Milan, Italy).

^1^H-NMR (300 MHz, CDCl_3_, 25 °C, TMS): δ 1.4 and 1.7 (m, 6H_PCL_ −[O(O)CCH_2_(**CH**_**2**_)_3_CH_2_]_122_−); δ 2.3 (2d, 2H_PCL_ −[O(O)CCH_2_(CH_2_)_3_**CH**_**2**_]_122_−); δ 2.7 (m, 4H_SA_ −C(O)(**CH**_2_)_2_C(O)−); δ 4.0 (t, 2H_PCL_ −[O(O)C**CH**_**2**_(CH_2_)_3_CH_2_]_122_−) (see Figure S1 in the Supporting Information).

### Synthesis and Characterization of PHEA-g-RhB-g-Succ-PCL

Derivatization of PHEA-g-RhB with PCL-Succ to obtain the PHEA-g-RhB-g-Succ-PCL graft copolymer was carried out under an argon atmosphere using CDI (as a condensing agent).^16^

Briefly, a calculated amount of CDI (*R*_3_ = mmol CDI/mmol PCL-Succ = 3) was added to the PCL-Succ dissolved in a-DMF (66.5 mg/mL). The solution was stirred at 40 °C for 5 h. Simultaneously, PHEA-g-RhB was dissolved in a-DMF (33 mg/mL) at 40 °C, and then, DEA was added as a catalyst (*R*_4_ = mmol DEA/[mmol repeating units (RUs) of PHEA-g-RhB] = 0.3). After the activation time, the resulting PHEA-g-RhB dispersion was added dropwise to CDI-activated PCL-Succ (*R*_5_ = mmol PCL-Succ/mmol RUs of PHEA-g-RhB = 0.12). The mixture was kept under continuous stirring at 40 °C for 68 h. Then, the reaction mixture was precipitated dropwise in diethyl ether; the obtained solid product was separated by centrifugation (at 4 °C for 15 min, at 9800 rpm) with a refrigerated centrifuge (Beckman) and washed several times with a diethyl ether/dichloromethane mixture (4:1 v/v). The obtained solid residue was dried and then dissolved in DMA, dialyzed against bidistilled water (MWCO 12–14 kDa), and freeze-dried.

^1^H-NMR (300 MHz, d_7_-DMF, 25 °C, TMS): δ 1.13 (m, 12H_RhB_**CH**_**3**_CH_2_−); δ 1.5 and 2.1 (m, 6H_PCL_ −[O(O)CCH_2_(**CH**_**2**_)_3_CH_2_]_122_−); δ 2.5 (2d, 2H_PCL_ −[O(O)CCH_2_(CH_2_)_3_**CH**_**2**_]_122_−); δ 2.7 (m, 4H_SA_ -C(O)(CH_2_)_2_C(O)-); δ 2.8 (m, 2H_PHEA_ −C(O)CH**CH**_**2**_C(O)NH−); δ 3.2 (t, 2H_PHEA_ −NH**CH**_**2**_CH_2_O−); δ 3.50 (t, 2H_PHEA_ −NHCH_2_**CH**_**2**_O−); δ 4.3 (t, 2H_PCL_ −[O(O)C**CH**_**2**_(CH_2_)_3_CH_2_]_122_−), and δ 5.0 (m, 1H_PHEA_ −NH**CH**(CO)CH_2_−); δ 7.00–8.00 (m, 10H_RhB_**H**-Ar) (see Figure S2 in the Supporting Information).

Size exclusion chromatography (SEC) analysis was done in 0.01 M LiBr DMF solution, as already reported (see Figure S3 in the Supporting Information).^16^

FT-IR analysis was also performed (see Figure S4 in the Supporting Information).

### Preparation of Empty and Roflumilast-Loaded Fluorescent Nanoparticles

Empty and Roflumilast-loaded fluorescent nanoparticles (named FNPs and FNPs@Roflumilast, respectively) were produced by nanoprecipitation.^17^ The PHEA-g-RhB-g-Succ-PCL graft copolymer dispersion (2 wt % in DMA) was placed in a buret and added dropwise to bidistilled water (organic/aqueous volume ratio equal to 3:20 v/v), at a flow rate equal to 1 mL/min. The mixture was left under stirring for 2 h and then purified by dialysis in bidistilled water (MWCO 12–14 kDa). The obtained NP dispersion was subsequently diluted to 1 mg/mL and filtered by a 5 μm cellulose acetate filter.

To produce FNPs@Roflumilast, Roflumilast (0.20 wt %), was dissolved in the DMA copolymer dispersion, placed in a buret, added dropwise to a saturated NaCl solution (3:20 v/v), and kept under stirring for 2 h. The obtained particles were purified by dialysis and centrifuged at 20 °C for 15 min at 8000 rpm, diluted to 1 mg/mL, and filtered by a 5 μm cellulose acetate filter. Both FNPs and FNPs@Roflumilast were then stored for further characterization.

### Preparation of Lipid Vesicles

Lipid vesicles were prepared using the thin lipid film method.^2^ Briefly, a mixture of DPPC/DSPE-PEG_2000_-mannose, at a molar ratio of 10:1, was dissolved in chloroform, which was evaporated under vacuum at 40 °C. The obtained lipid film was rehydrated using 25 mL of bidistilled water (to obtain a final lipid concentration of 0.56 wt %) and stirred in a water bath at 75 °C for 30 min. Furthermore, the same procedure was followed using DPPC in chloroform to produce untargeted lipid vesicles to be used as controls.

### Preparation of Lipid–Polymer Hybrid Fluorescent Nanoparticles (LPHFNPs)

The two-step method was carried out to prepare empty and Roflumilast-loaded mannosylated LPHFNPs (named Man-LPHFNPs and Man-LPHFNPs@Roflumilast, respectively). In particular, the dispersions of FNPs and liposomes were mixed together (polymer/lipid phase ratio of 3:2 v/v), and the physical mixture was subjected to high-pressure homogenization (HPH) at a pressure of 10,000–15,000 psi using an EmulsiFlex-C5 homogenizer. Each obtained dispersion was purified by ultracentrifugation (Optima XPN Ultracentrifuge, Type 70 Ti Rotor) at RT for 1 h at 40,000 rpm, and the pellet was stored for further characterization.

Furthermore, to produce empty and Roflumilast-loaded LPHFNPs (LPHFNPs and LPHFNPs@Roflumilast, respectively), to be used as controls, the same procedure was followed starting from FNPs and DPPC-based vesicles.

### Characterization of Man-LPHFNPs

#### Dimensional Analysis and ζ-Potential Measurements

The mean size (nm) and polydispersity index (PDI) of obtained samples were determined in bidistilled water or 10 mM NaCl aqueous solution by dynamic light scattering (DLS) using a Malvern Zetasizer NanoZS (Malvern Instruments, Worcestershire, U.K.) instrument equipped with a 632.8 nm laser with a fixed scattering angle of 173° using Dispersion Technology Software 7.02.

Zeta potential measurements were performed by electrophoresis measurements, in bidistilled water or 10 mM NaCl aqueous solution, at 25 °C using the same apparatus. ζ Potential values (mV) were calculated from electrophoretic mobility using the Smoluchowski relationship. Analyses were performed in triplicate.

#### Scanning Transmission Electron Microscopy (STEM)

The Man-LPHFNP morphology was determined by scanning transmission electron microscopy (STEM). To prepare the STEM grids, one drop of sample dispersion (3 mg/mL) was placed on a holey carbon-coated copper grid, air-dried overnight, and imaged using an SEM/STEM Fei-ThermoFisher Versa 3D.

#### Phospholipid Quantification

The lipid content in Man-LPHFNPs (or LPHFNPs as control samples) was evaluated using the colorimetric assay of ammonium ferrothiocyanate.^18^ A proper amount of each sample was dispersed in chloroform, and the obtained dispersion was placed in contact with an ammonium ferrothiocyanate aqueous solution. The obtained mixture was shaken for 15 min; then, the organic layer was separated, and its absorbance was measured at λ = 488 nm by an RF-5301PC spectrofluorometer (Shimadzu, Italy). Each experiment was repeated at least three times. A calibration curve was obtained using mixtures of DPPC/DSPE-PEG_2000_-mannose at concentrations ranging between 0.1 and 0.005 mg/mL (*y* = 6.4049x, *R*^2^ = 0.998).

#### X-ray Photoelectron Spectroscopy (XPS) Analysis

XPS spectra were recorded on each freeze-dried sample using a PHI 5000 VersaProbe II (ULVAC-PHI, Inc., Chigasaki, Japan) and monochromatic Al-Kα radiation (hϖ = 1486.6 eV) with a 128-channel hemispheric analyzer, FAT mode. The acquisition conditions were as follows: high resolution (C 1s, N 1s, P 2p regions): step 0.0500 eV; time per step 50 ms; X-ray source with beam ⌀ 200 μm, 50 W, 15 kV; 45° angle, Pass Energy 23,500 eV; 20 min/region; charge compensation with Ar + and e–. The fitting was done using the model Gauss–Lorentz, Shirley background, with the software Multipak 9.9.0 (ULVAC-PHI, Inc.).

#### Determination of Surface PEG Density and PEG Chain Conformation on the Man-LPHFNP Surface

The surface PEG density of Man-LPHFNPs and the chain conformation were determined by an ^1^H-NMR method reported elsewhere.^19^ First, a calibration curve for the PEG signal at δ 3.6 ppm, starting from PEG solutions in D_2_O, in concentrations ranging between 0.5 and 2 mg/mL (*y* = 1.3007x, *R*^2^ = 0.982), was obtained using RhB (4 mg/mL) as the internal standard. Man-LPHFNPs were then suspended in D_2_O (16.6 mg/mL), and spectra were acquired, repeating each determination in triplicate. The LPHFNPs were also analyzed as control samples.

### Measurement of Interactions between Man-LPHFNPs and Mucin

#### Turbidimetric Assay

The interactions between Man-LPHFNPs (or LPHFNPs as control samples) and mucin were determined by turbidimetry.^13,19^ Briefly, we prepared dispersions of LPHFNPs or Man-LPHFNPs (0.2 mg/mL) and mucin (2 mg/mL) in PBS. Then, equal volumes of each dispersion were mixed and kept under magnetic stirring for 1 min. After incubation at 37 °C, the transmittance was measured at 0, 50, 100, 150, 200, 250, and 300 min, at λ of 650 nm by a UV spectrophotometer. Results were expressed as % transmittance compared to the transmittance values calculated for the mucin. All of the experiments were run in triplicate.

#### Mucus Model

The mucus model was prepared as previously described.^12^ Briefly, 250 mg of mucin, 0.295 mg of DTPA, 1 mL of RPMI 1640 amino acid solution, 250 μL of egg yolk emulsion, 250 mg of NaCl, 110 mg of KCl, and 1.5 wt % HEC were mixed together in a final volume of 50 mL of bidistilled water. This dispersion was allowed to equilibrate at 25 °C for 2 h.

#### Rheological Analysis

Rheological properties of artificial mucus alone or in the presence of Man-LPHFNPs (or LPHFNPs as control samples) at a concentration of 0.1 mg/mL were determined at 20 °C using a rotational rheometer (TA Instruments) coupled to 8 mm parallel plate geometry and a controlled Peltier plate, maintaining a gap of 300 μm. First, the linear viscoelastic region of artificial mucus at 1 wt % HEC was determined by a strain sweep test ranging from 0.01 to 20%, which was found to be in the range of 5–10%. Then, a frequency sweep (0.01–2 Hz) was performed for all samples at 5% constant strain to determine the complex viscosity (η*). All rheological tests were conducted in triplicate, and Trios Software v3.3 TA Instruments was used for data acquisition and analysis.

#### Determination of Drug Loading (DL%)

The amount of entrapped Roflumilast loaded into the samples was expressed as drug loading (DL%), which is the weight percent ratio between the drug and the sample. It was determined by HPLC analysis using a Waters Breeze System liquid chromatograph equipped with an autosampler (40 μL injection volume) and a Shimadzu UV–vis HPLC detector. Analyses were performed using a mobile phase of acetonitrile/acid water at pH 3 (60/40, v/v) with a 1 mL/min flow, taking UV readings at a wavelength of 250 nm. The chosen column (Luna C18 100A, 250 × 4.6 mm, from Phenomenex) was equilibrated to 25 °C. Roflumilast was extracted by dispersing a known amount of each sample in 200 μL of DMA to which 1.3 mL of acetonitrile was subsequently added. The obtained dispersion was filtered (PTFE filter, 0.45 μm) prior to HPLC analysis.

The obtained peak area at 9.60 min was compared with a calibration curve obtained by plotting areas versus standard solution concentrations of Roflumilast in the range of 0.025–0.1 mg/mL (*y* = 1 × 10^8^ ×, *R*^2^ = 0.999).

#### Roflumilast Release from Man-LPHFNPs

The Roflumilast release profile from LPHFNPs was realized using the dialysis method, under sink conditions. Briefly, a known amount of Man-LPHFNPs@Roflumilast (or LPHFNPs@Roflumilast as the control sample) was dispersed in 1 mL of PBS at pH 7.4, placed in a dialysis tubing (MWCO 12–14 kDa), immersed in 30 mL of PBS (pH 7.4)/ethanol (80/20, v/v), and then incubated at 37 °C under continuous stirring in an orbital shaker. Then, 0.5 mL of the external medium was withdrawn and replaced with an equal volume of fresh medium, at fixed time intervals (1, 2, 4, 6, and 24 h). Samples were freeze-dried, and Roflumilast was extracted by dissolving the obtained powder in 5 mL of acetonitrile. The obtained dispersions were then purified by centrifugation, filtered (PTFE filter, 0.45 μm), and injected using the HPLC method described for DL% determination. The dissolution profile of Roflumilast was also evaluated under the same experimental conditions.

#### Differential Scanning Calorimetry (DSC) Analysis

Differential scanning calorimetric (DSC) analysis was performed with a DSC 131 Evo (Setaram). Each sample (LPHFNPs, liposomes, or FNPs) was sealed in an aluminum pan and submitted to heating and cooling cycles in the temperature range of 20–300 °C at a scanning rate of 5 °C/min for heating and a scanning rate of 10 °C/min for cooling. Each analysis was carried out on 5–10 mg of sample weight.

### Biological Characterization

#### Cell Cultures

Human bronchial epithelial cells (16-HBE) were furnished by Istituto Zoo-profilattico of Lombardia and Emilia Romagna. The line of murine macrophage RAW 264.7 cells was purchased from ATCC (Manassas, VA). Cells were grown in a minimum essential medium [Dulbecco’s modified Eagle’s medium (DMEM)] supplemented with 10 vol % fetal bovine serum (FBS), 2 mM l-glutamine, 100 U/mL penicillin, 100 μg/mL streptomycin, and 2.5 μg/mL amphotericin B (all reagents were from Euroclone, Milan, Italy) under standard conditions (95% relative humidity, 5% CO_2_, 37 °C). Cells were allowed to grow until confluence, trypsinized, and seeded in plates at 2.5 × 10^4^ cells/cm^2^ (tissue culture grade, 96 wells, flat bottom) for each experiment of cell viability or cell uptake.

#### MTS Cell Viability Assay

Cell viability was assessed by an MTS assay, using a commercially available kit (CellTiter 96 Aqueous One Solution Cell Proliferation assay, Promega). In particular, 16-HBE and RAW 264.7 were incubated with 200 μL per well with an aqueous dispersion (DMEM containing 10% FBS) of Roflumilast free or loaded into Man-LPHFNPs (or LPHFNPs as control samples), to obtain drug concentrations ranging between 0.45 and 0.112 μg/mL. Cell viability was also evaluated in the presence of empty LPHFNPs at concentrations corresponding to those of drug-containing systems. All dispersions were sterilized by filtration using a 220 nm filter.

After 24 h of incubation, the supernatant was removed and each plate was washed with sterile DPBS; after this, cells in each well were incubated with 100 μL of fresh DMEM and 20 μL of an MTS solution, and plates were incubated for 2 h at 37 °C. The absorbance at 490 nm was read using a Microplate reader (Multiskan Ex, Thermo Labsystems, Finland). Relative cell viability (percentage) was expressed as (Abs490 treated cells/Abs490 control cells) × 100, on the basis of three experiments conducted in multiples of six. Cells incubated with the medium were used as negative controls.

#### Cell Uptake

RAW 264.7 and 16-HBE were seeded at 3.0 × 10^4^ cells/cm^2^ and treated the day after with 200 μL of Man-LPHFNP (or LPHFNPs as control samples) dispersion in DMEM (0.125 mg/mL). For the inhibition assay, 10 mM mannose was added to the incubation medium prior to adding samples. The control experiment was conducted by incubating cells with 200 μL of DMEM.

After incubation at 37 °C for 4 and 24 h, the cells were washed, fixed with 4 vol % paraformaldehyde in PBS at room temperature, and treated with the 4,6-diamidino-2-phenylindole (DAPI) fluorescent dye (Thermo Scientific, Waltham, MA) for 5 min to stain the nuclei. The images were acquired using an inverted epifluorescence microscope analyzed by the use of AxioVision software. Experiments were carried out in triplicate for each incubation time.

#### Preparation and Characterization of NiM Formulations

NiM formulations were prepared using a Nano Spray Dryer B-90 (Buchi, Milan, Italy). First, a mixture of PVA and Leu was solubilized in a water/ethanol dispersion (9:1 v/v), at a concentration of 2.5 wt/v%, in a weight ratio of 75:25, respectively. After the complete dissolution, a proper amount of freeze-dried Man-LPHFNPs@Roflumilast was added (0.5 wt/v% concentration). The obtained mixture was filtered with a 5.0 μm cellulose acetate filter and subsequently spray-dried, with a spray cap of 4.0 μm at the inlet temperature of 92 °C. Filtered and dehumidified air was used as the drying gas, at a flow rate of 120 L/min (resulting in an inside pressure of 38 mbar at a spray rate of 78%). The obtained NiM were collected and analyzed by SEM analysis (PRO X PHENOM, Thermo Fisher Scientific, Milan, Italy), using the ImageJ program to calculate the average diameter of each sample by analyzing a sufficiently representative number of particles (>500 particles). The NiM powder was also dispersed in water and 10 mM NaCl aqueous solution, and the obtained dispersion was analyzed by DLS.

#### Statistical Analysis

All experiments were repeated at least three times. All data are expressed as means ± SD. All data were analyzed by Student’s *t*-test. A *P*-value < 0.05 was considered statistically significant, a *P*-value < 0.001 was considered highly significant, whereas a *P*-value > 0.05 was considered not statistically significant.

## Results and Discussion

### Preparation and Characterization of Lipid–Polymer Hybrid Nanoparticles (LPHFNPs)

In this work, we produced empty mannosylated or Roflumilast-loaded mannosylated LPHFNPs (Man-LPHFNPs or Man-LPHFNPs@Roflumilast, respectively) following a two-step method by properly combining preformed fluorescent polymeric nanoparticles and preformed lipid vesicles.

**Scheme 1:**
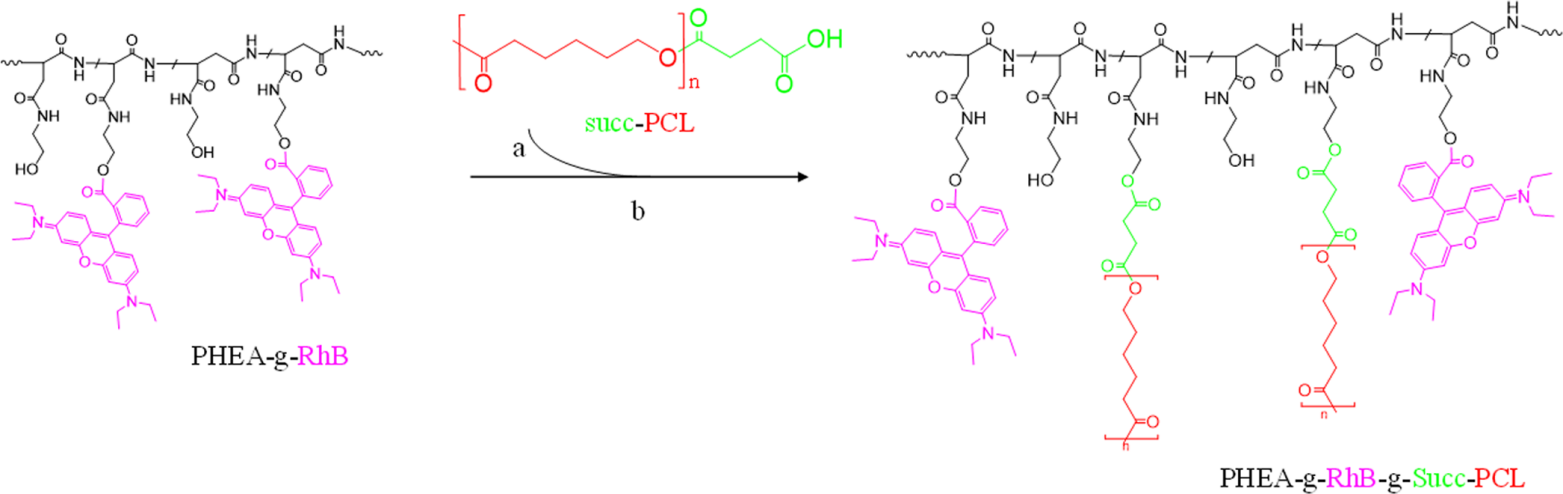
Synthetic Route of the PHEA-g-RhB-g-Succ-PCL Graft Copolymer: PHEA (Black), RhB (Fuchsia), PCL (Red, *n* = 122), Succinic Residue (Succ, Green) Reagents and conditions: (a) a-DMF, CDI, DEA, 5 h at 40 °C; (b) 68 h at 40 °C.

In particular, empty or Roflumilast-loaded fluorescent nanoparticles (named, respectively, FNPs and FNPs@Roflumilast) were prepared by nanoprecipitation starting from a fluorescent polyaspartamide-polycaprolactone graft copolymer, without the use of surfactants or any stabilizing agents thanks to the amphiphilic properties of the copolymer.^12^ The latter is a biocompatible and biodegradable graft copolymer synthesized starting from α,β-poly(*N*-2-hydroxyethyl)-dl-aspartamide (PHEA), whose derivatives have been widely used for drug and gene delivery applications.^12,20^ On the PHEA backbone were covalently linked appropriate quantities of RhB (to make fluorescent the resulting derivates) and a succinate-polycaprolactone derivate, the PCL-Succ, to obtain an amphiphilic graft copolymer, the PHEA-g-RhB-g-Succ-PCL, which is highly processable and useful in obtaining polymeric nanoparticles by nanoprecipitation.^15,16^ To increase the reactivity of PCL toward the coupling reaction with PHEA hydroxyl groups, the PCL hydroxyl group was previously succinylated with SA, obtaining the PCL-Succ (see Figure S1 in the Supporting Information for the ^1^H-NMR spectrum). The synthetic scheme of the PHEA-g-RhB-g-Succ-PCL graft copolymer is reported in Scheme 1.

The degree of derivatization in PCL (DD_PCL_), determined from the ^1^H-NMR spectrum (see Figure S2 in the Supporting Information for the ^1^H-NMR spectrum), was equal to 2.9 ± 0.3 mol %.^16^ FT-IR analysis and size exclusion chromatography (SEC) confirmed the occurrence of the conjugation reaction of PCL-Succ on PHEA-RhB, with the *M̅*_w_ being equal to 136 kDa (*M̅*_w_/*M̅*_n_ = 1.50). Moreover, compared to the nonlabeled polymer, whose synthesis has been described elsewhere, there are no significant differences on the *M̅*_w_ and the DD_PCL_, with both cases having about 6 and 7 chains for each PHEA chain.^16^ The obtained polydispersity value could be due to the experimental conditions in which the SEC analysis was carried out in order to analyze in the same conditions all of the materials, which are PCL-Succ, PHEA-g-RhB, and PHEA-g-RhB-g-Succ-PCL copolymers; in any case, a separation procedure could be applied to select a narrower range of molecular weights before its use in the clinic. The SEC chromatogram and the FT-IR analysis of the obtained copolymer are reported in Figures S3 and S4, respectively, in the Supporting Information.

Being lipophilic, Roflumilast was successfully entrapped during the nanoparticle formation, by solubilizing it in the copolymer organic dispersion. In addition, the use of a saturated NaCl solution instead of bidistilled water, as an external phase, resulted in a higher encapsulation efficiency of Roflumilast, with the drug loading % (DL%) being equal to 1.0 ± 0.2%.

After proper purification by dialysis and centrifugation, the obtained samples were characterized in terms of the mean size, PDI, and ζ potential, and the results are reported in Table 1.

**Table 1:** Mean Size, Polydispersity Index (PDI), and ζ Potential Values of FNPs and FNPs@Roflumilast^a^

sample	mean size (nm ± S.D.)	PDI ± S.D.	ζ potential (mV ± S.D.)
FNPs	59.0 ± 3.1	0.21 ± 0.12	–6.4 ± 5.4
FNPs@Roflumilast	78.0 ± 4.1	0.24 ± 0.07	–8.2 ± 4.3

a Data are expressed as means ± SD (*n* = 3).

FNPs showed a high size uniformity and a mean size of about 59 nm, while the presence of entrapped Roflumilast affects this value, with the diameter of FNPs@Roflumilast being equal to 78 nm. ζ -Potential values of both samples were slightly negative and did not significantly change after drug incorporation, suggesting the absence of drug on the nanoparticle surface.

Lipid vesicles were produced by the thin-layer method and by choosing 1,2-dipalmitoyl-*sn*-glycero-3-phosphocholine (DPPC) and 1,2-distearoyl-*sn*-glycero-3-phosphoethanolamine-*N*-(polyethylene glycol)_2000_-mannose (DSPE-PEG_2000_-mannose) as starting lipid materials. DPPC was chosen because it is the main phospholipid in the human lung surfactant; therefore, it was very well tolerated by the body.^10^ It is a well-characterized endogenous lipid, widely used to prepare liposomes and LPHFNPs for drug delivery.^21,22^ Thanks to its zwitterionic charge, DPPC can ensure the formation of an adequate ζ-potential, stabilizing the resulting LPHFNPs and preventing their aggregation.^2^

To ensure targeted delivery of Roflumilast to alveolar macrophages, DSPE-PEG_2000_-mannose was mixed with DPPC to produce phospholipid vesicles. Mannosylation represents a suitable strategy to obtain an active targeting to macrophages because of the overexpression of mannose receptors (CD-206, CD-163, and CD-204) on the surface of alveolar macrophages in the lungs of patients with COPD.^11,23^

Therefore, Man-LPHFNPs and Man-LPHFNPs@Roflumilast were prepared by high-pressure homogenization (HPH), starting from a mixture between two dispersions, one containing FNPs@Roflumilast and the other containing the mixture of DPPC/DSPE-PEG_2000_-Mannose-based vesicles. Following the same procedure, we prepared empty and Roflumilast-loaded LPHFNPs based on DPPC (LPHFNPs and LPHFNPs@Roflumilast, respectively) to be used as control samples for further characterizations.

After proper purification, all obtained LPHFNPs were characterized by DLS analysis in bidistilled water. Data are reported in Table 2.

**Table 2:** *Z*-Average Size, Polydispersity Index (PDI), and ζ Potential Values of LPHFNPs, before and after Freeze-Drying in Bidistilled Water, and after Freeze-Drying in a 10 mM NaCl Solution^a^

sample	*Z*-average (nm ± S.D.)	PDI ± S.D.	ζ Potential (mV ± S.D.)
**Before Freeze-Drying/water**			
LPHFNPs	82.8 ± 3.2	0.22 ± 0.01	–8.8 ± 5.7
LPHFNPs@Roflumilast	110.8 ± 3.5	0.20 ± 0.11	–9.9 ± 4.5
Man-LPHFNPs	72.1 ± 4.2	0.16 ± 0.04	–21.4 ± 6.4
Man-LPHFNPs@Roflumilast	94.0 ± 1.3	0.15 ± 0.04	–20.0 ± 5.9
**After Freeze-Drying/water**			
LPHFNPs	91.4 ± 4.5	0.17 ± 0.01	–8.7 ± 6.2
LPHFNPs@Roflumilast	114.6 ± 2.6	0.19 ± 0.03	–9.6 ± 6.4
Man-LPHFNPs	88.5 ± 4.1	0.22 ± 0.08	–18.8 ± 6.4
Man-LPHFNPs@Roflumilast	114.5 ± 1.6	0.20 ± 0.02	–15.0 ± 5.1
**After Freeze-Drying/NaCl 10 mM**			
LPHFNPs	169.8 ± 2.2	0.22 ± 0.04	–7.4 ± 4.5
LPHFNPs@Roflumilast	222.4 ± 4.5	0.28 ± 0.10	–9.6 ± 4.9
Man-LPHFNPs	159.1 ± 3.2	0.24 ± 0.03	–7.5 ± 4.9
Man-LPHFNPs@Roflumilast	155.0 ± 1.7	0.25 ± 0.06	–10.8 ± 5.6

a Data are expressed as means ± SD (*n* = 3).

The chosen experimental conditions allowed us to obtain empty LPHFNPs with a mean size, expressed as *Z*-average, ranging between 70 and 80 nm, influenced by the entrapped drug, as the *Z*-average increases in the presence of Roflumilast until 94–110 nm. Moreover, all samples showed narrow dimensional distribution, with the PDI being below 0.22.

To evaluate how the drying process could affect the aqueous redispersion of LPHFNPs, DLS analysis was repeated after freeze-drying. The data obtained suggest that it mildly influences particle aggregation, with the mean size values of obtained systems increased by about 15% more than that obtained before the freeze-drying process.

ζ Potential values in bidistilled water, reported in Table 2, are near neutral before and after the freeze-drying of LPHFNPs and become higher and more negative in the mannosylated samples. This charge increase shift can be attributed to the presence of mannose on the nanoparticle surface, as already reported for other mannosylated carriers.^24^ In addition, there are no significant differences between empty and Roflumilast-loaded samples.

Although the presence of phospholipids on the nanoparticle surface could stabilize the system, preventing aggregation, several other factors, such as ionic strength, can affect the colloidal stability.^25^ As already reported elsewhere, the increase in ionic strength from 1 to 150 mM leads to an aggregation of LPHFNPs by screening their electrostatic charges.^2,22^

To evaluate the effect of ionic strength of the aqueous phase on the colloidal stability of our LPHFNPs, we carried out a DLS analysis in a 10 mM NaCl solution on freeze-dried samples.^26^

From the data, also reported in Table 2, all samples showed mean size and PDI values slightly higher than those obtained in bidistilled water, demonstrating that the presence of the salt favors some aggregation phenomena, however, in the colloidal range. Moreover, in a saline solution, a reduction in ζ potential values was obtained for mannosylated samples as compared to those obtained in bidistilled water.

In Figures S5–S8 of the Supporting Information, one of the three distribution curves of both the mean size and the ζ potential, obtained, respectively, from the samples LPHFNPs, LPHFNPs@Roflumilast, Man-LPHFNPs, and Man-LPHFNPs@Roflumilast, before freeze-drying (a), and after freeze-drying and redispersion in water (b) or in 10 mM NaCl aqueous solution (c), are reported. As can be clearly seen, there is a single particle population system in each sample, confirming the formation of hybrid systems consisting of polymer nanoparticles embedded within the lipid layer.

**Figure 1:**
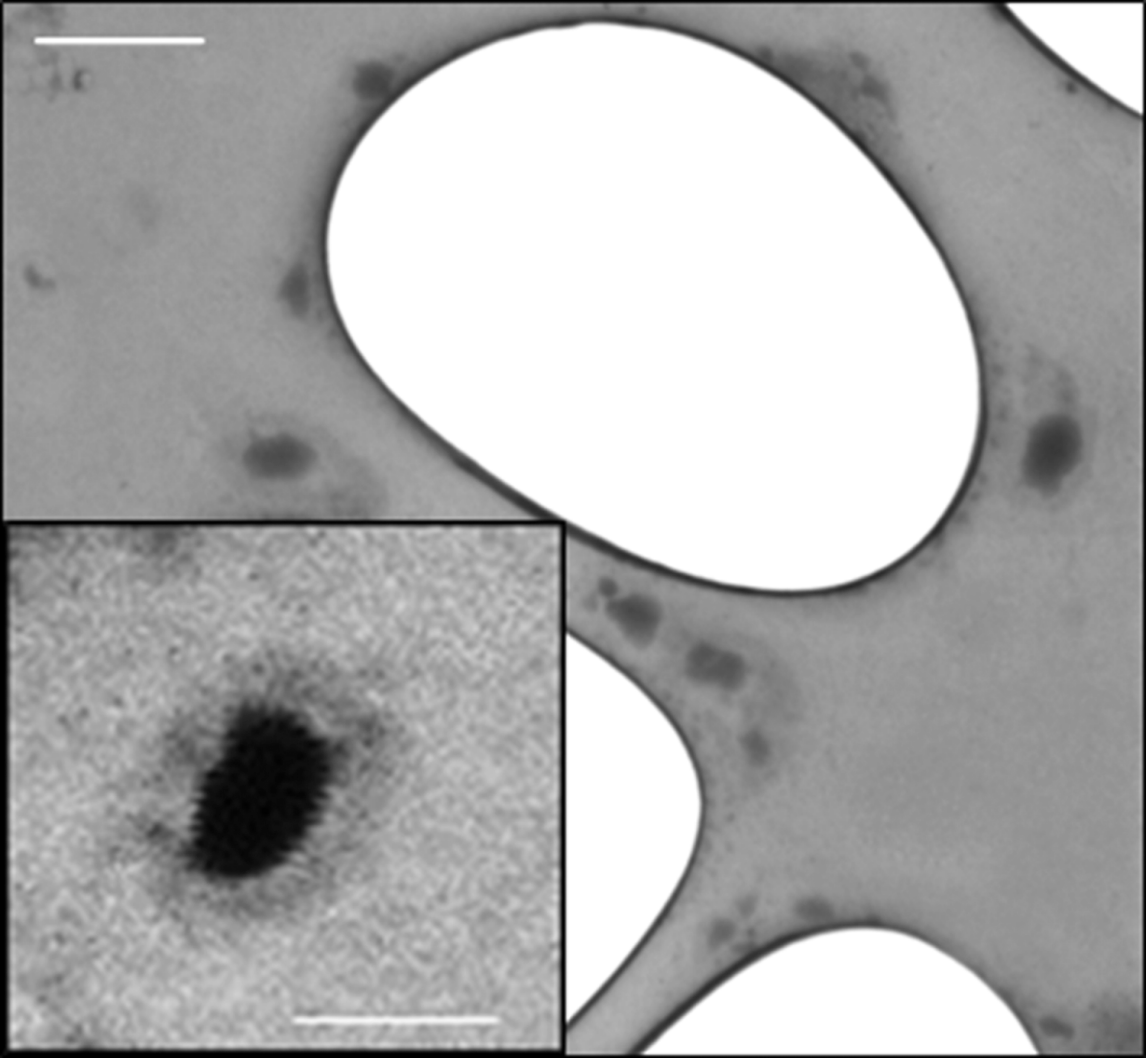
STEM image of Man-LPHFNPs. The bar represents 200 nm. One drop of the sample dispersion (3 mg/mL) was placed on a holey carbon-coated copper grid, air-dried overnight, and imaged using an SEM/STEM Fei-ThermoFisher Versa 3D.

To obtain morphology information, the Man-LPHFNP sample was subjected to a transmission and scanning (STEM) analysis. Recorded images are shown in Figure 1 for the Man-LPHFNP sample. As can be seen in the magnification, the particles exhibited a spherical and nanosized dimension, with a core–shell structure, having an outer bright portion, attributed to the lipid shell, and a darker core, attributed to the polymeric portion.

The lipid content in each sample was determined by the colorimetric assay of ammonium ferrothiocyanate and is reported in Table 3.^18^ This method, developed for the quantification of phospholipids in biological samples, is based on the formation of a complex between phospholipids and the ammonium ferrothiocyanate, which is quantifiable by UV analysis at λ = 488 nm.

**Table 3:** Phospholipid Content for Each LPHFNP Sample, Determined by the Ammonium Ferrothiocyanate Colorimetric Assay^a^

lipid content (wt % ± S.D.)	
LPHFNPs	46.82 ± 6.33
LPHFNPs@Roflumilast	44.42 ± 5.03
Man-LPHFNPs	44.65 ± 2.33
Man-LPHFNPs@Roflumilast	50.83 ± 0.86

a Data are expressed as means ± SD (*n* = 3).

Results, expressed as the percent weight ratio between the lipid amount and the total weight of the sample, indicated that all of the samples contain about 44–51 wt % of the lipids, with no statistically significant differences.

**Figure 2:**
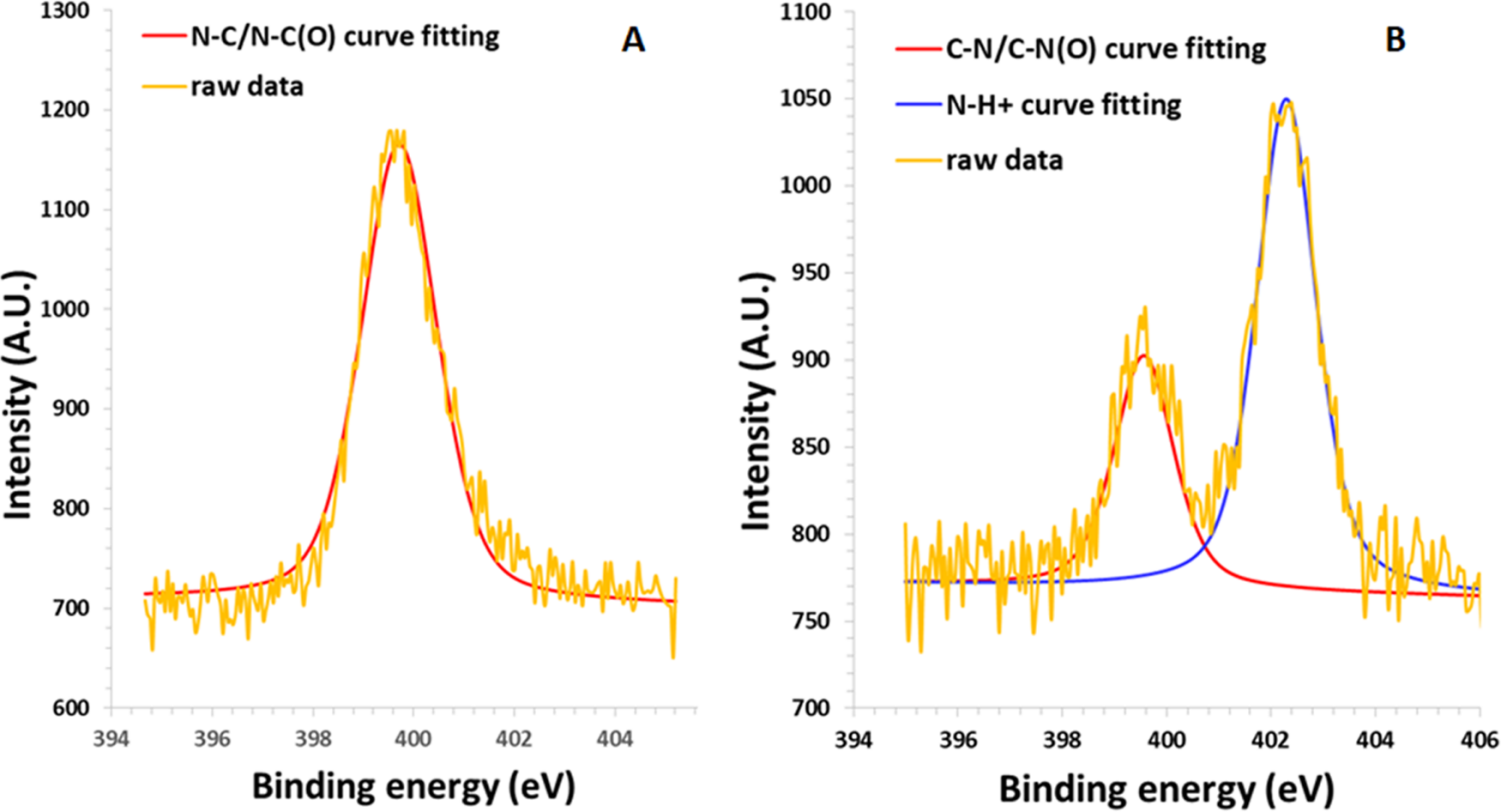
XPS curve-fitting of the photoelectron peak N 1s of (A) FNPs and (B) Man-LPHFNPs. Spectra were recorded on each freeze-dried sample using a PHI 5000 VersaProbe II and monochromatic Al-Kα radiation (*h*v = 1486.6 eV) from an X-ray source operating at a spot size of 200 μm, a power of 50 W, and an acceleration voltage of 15 kV. The fitting was done using the model Gauss–Lorentz, Shirley background. The scattered yellow line refers to the raw data, while the solid red and blue lines refer to the curve-fitting results.

Subsequently, to confirm the presence of phospholipids on the surface, an XPS analysis was carried out, on both Man-LPHFNPs and FNPs (as control samples). Data, expressed as relative distribution of the carbon, nitrogen, oxygen, and phosphorus species on the sample surface, determined by a curve-fitting procedure of the photoelectron signals, are reported in Table 4, whereas the curves of fittings of the N 1s spectra are reported in Figure 2.

**Table 4:** XPS Surface Chemical Composition of FNPs and Man-LPHFNPs Expressed as Relative Distribution of C, N, O, and P

sample	C 1s	N 1s	O 1s	P 1s
FNPs	69.7	3.9	26.4	0
Man-LPHFNPs	80.8	2.3	14.9	1.8

**Figure 3:**
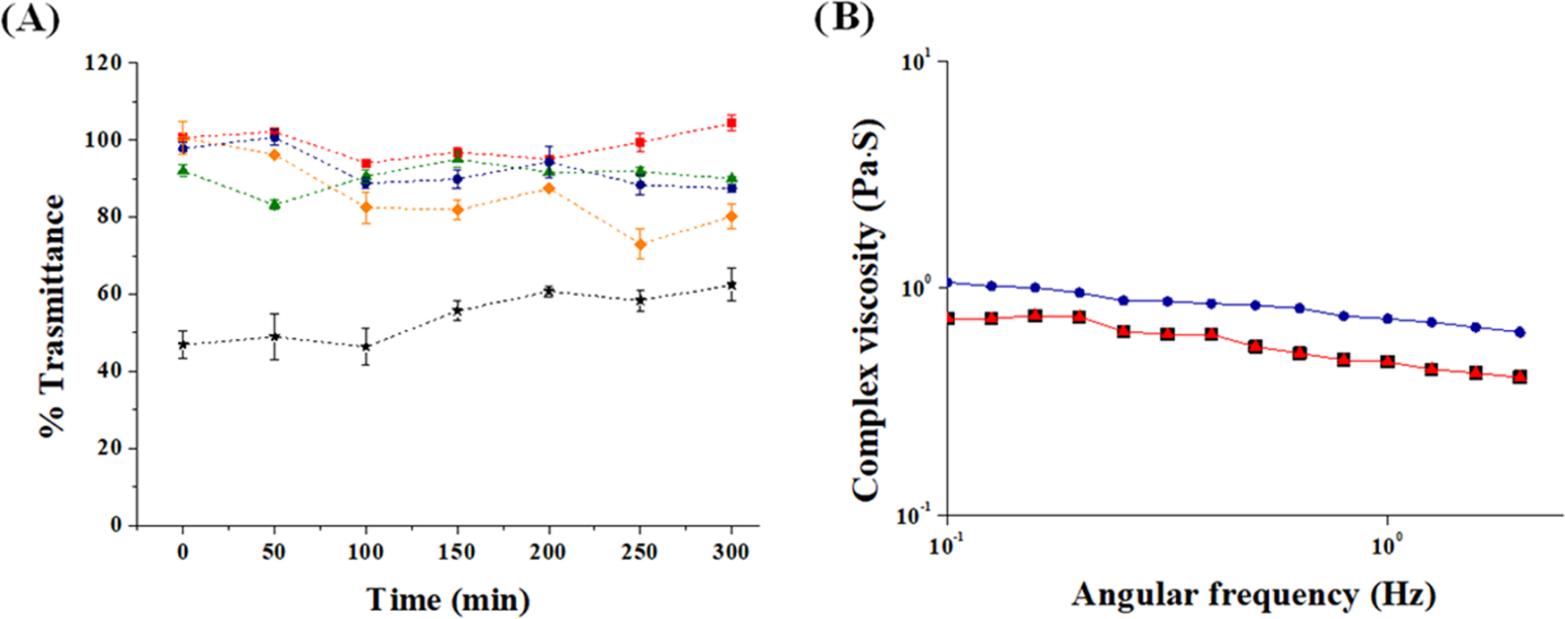
(A) Transmittance at 650 nm of dispersions containing mucin in the presence of Man-LPHFNPs [0.1 mg/mL blue; 0.5 mg/mL orange], LPHFNPs [0.1 mg/mL red; 0.5 mg/mL green], and chitosan [0.5 mg/mL, black]. (B) Complex viscosity of artificial mucus alone (black) and treated with LPHFNPs 0.1 mg/mL (red) and Man-LPHFNPs 0.1 mg/mL (blue).

As can be seen, among the elements found on the surface of the Man-LPHFNP sample, there is phosphorus, compatible with the phospholipid structure, which is absent in the FNP sample based on the PHEA-g-RhB-g-Succ-PCL graft copolymer. Moreover, the N 1s spectrum of FNPs, reported in Figure 3 A, shows a single peak at 399.7 eV, attributed to amidic nitrogen highly abundant in the PHEA-based copolymer. On the other hand, the N 1s spectrum of Man-LPHFNPs, reported in Figure 3B, shows two peaks: one at 399.7 eV, of about 32.1%, and another at 402.3 eV, attributed to the N–H^+^ bond, which is predominant, accounting for 67.9% of the total nitrogen. This result confirmed the presence of phospholipids on the Man-LPHFNP surface.

Although the previous characterizations demonstrate that phospholipids are on the LPHFNP surface, no indications allowed us to know if the PEG (and therefore the mannose at the end of the chain) was on the LPHFNP surface. For this reason, we evaluated the amount of PEG moieties on the surface of Man-LPHFNPs using a method based on ^1^H-NMR analysis, already reported elsewhere.^19^ LPHFNPs were analyzed as the control sample. By comparing the integrals of the PEG peak in the spectrum of the Man-LPHFNP aqueous dispersion to the PEG calibration curve, the surface pegylation was found to be equal to 0.00297 mmol/100 mg Man-LPHFNPs, corresponding to about 70 wt % of the total DSPE-PEG_2000_-mannose amount entrapped into the core–shell structure of LPHFNPs.

Hence, by assuming all surface PEG chains were full length with 2 kDa PEG, the surface PEG density [Γ] (number of PEG per 100 nm^2^) was calculated using a formula reported elsewhere.^19^ In particular, the [Γ] obtained value of about 30 molecules of PEG per 100 nm^2^ demonstrated a high surface PEG density. However, the high degree of surface pegylation does not assure the exposition of the mannose molecule linked at the end of each PEG chain, which is necessary either to increase the interaction of the targeting moiety to the CD-206 receptors present on the surface of alveolar macrophages or to ensure particle mucodiffusion through the mucus layer in pathological lung conditions.^27^ Several studies have already suggested that coating the surface of nanoparticles with PEG, with a molecular weight sufficiently low to avoid mucoadhesive interactions with mucins, and also with an adequate surface density, can enhance their diffusion into the mucus layer.^28^

For PEG moieties with a *M̅*_w_ of 2000 Da, the number of unconstrained chains that occupy 100 nm^2^ of the particle surface area [Γ*] was found to be 11.025.^19^ We calculated [Γ/Γ*], which is an index necessary to deduce the assumed conformation of the PEG chains on the surface; [Γ/Γ*] for Man-LPHFNPs was found to be 2.72, indicating a high surface PEG density and a brushlike conformation, with long, thin bristles of PEG extending from the LPHFNP surface.^19^ This brushlike conformation is necessary for imparting mucus penetration properties to nanoparticles; as reported elsewhere, the traditional “brush” PEG corona would facilitate the penetration of the pegylated particles through the mucus layer.^29^ On the contrary, pegylated NPs with a loop conformation (mushroom) would increase the time of residence of the adhered fraction of particles on the mucus layer.

Other polymeric nanosystems, with similar characteristics compared to ours in terms of size, zeta potential, surface PEG density, and PEG chains conformation, have already demonstrated a high capacity to penetrate the mucus layers.^29^

To evaluate whether the resulting surface pegylation degree and conformation of LPHFNPs could effectively reduce interactions with mucin present in the lung mucus, a turbidimetric assay was carried out.

In particular, mucoadhesive nanoparticle–mucin interactions determine aggregation due to the adsorption process of mucin on the nanoparticles, quantified as a reduction in transmittance over time.^30^ The turbidity of mucin/Man-LPHFNPs and mucin/LPHFNPs aqueous dispersions at two different concentrations was measured by UV analysis over 6 h and compared to that of a mucin/chitosan dispersion as the positive control. The obtained data are reported in Figure 3A as transmittance values at 650 nm as a function of the incubation time. Both samples, at a concentration of 0.1 mg/mL, do not give unfavorable interactions with mucin, as evidenced by transmittance values close to 100% over 6 h. On the other hand, it is possible to observe how the transmittance of Man-LPHFNPs, at a concentration of 0.5 mg/mL, decreases (less than 80%), after about 3 h, suggesting a slight mucoadhesion of the sample. In contrast, the positive control chitosan leads to a significant decrease in transmittance over 6 h of incubation.

To obtain more information on particle–mucin interactions, a rheological study was also carried out. In particular, an artificial mucus model was used, prepared following a previously described method.^12^ As already known, this mucus shows a rheological behavior typical of pseudoplastic fluids, with a viscosity reduction after shear stress increase, similar to that observed for sputum of COPD patients.^31,32^ Accordingly, to carry out our experiment, artificial mucus was incubated alone or in the presence of LPHFNPs and Man-LPHFNPs, and the complex viscosity (η*) was determined by a rotational rheometer (Figure 3B).

As shown, no interaction occurs between the LPHFNPs and mucus, as the complex viscosity of the mixture superimposed is comparable to that of mucus alone. On the other hand, at the same shear stress applied, the mannosylated LPHFNPs lead to a slight increase in the complex viscosity compared to both mucus alone and in the presence of LPHFNP, confirming the results obtained from the turbidimetric study. Therefore, Man-LPHFNPs showed some capacity to interact with the components of the mucus, under the chosen experimental conditions, which could be attributed to the presence of mannose residues exposed on the LPHFNP surface.

Once realized and characterized, the amount of entrapped Roflumilast in Man-LPHFNPs@Roflumilast and LPHFNPs@Roflumilast was evaluated by HPLC analysis and expressed as drug loading (DL%) (Table 5). As can be seen, starting from the same FNPs@Roflumilast, comparable LPHFNPs were obtained, with both the DL% and the EE% not significantly different (*p* > 0.05).

**Table 5:** Drug Loading (% wt) of LPHFNPs Containing Roflumilast

sample	dl% (wt % ± S.D.)	EE% (wt % ± S.D.)
LPHFNPs@Roflumilast	0.24 ± 0.03	10.50 ± 2.5
Man-LPHFNPs@Roflumilast	0.28 ± 0.07	13.52 ± 3.2

**Figure 4:**
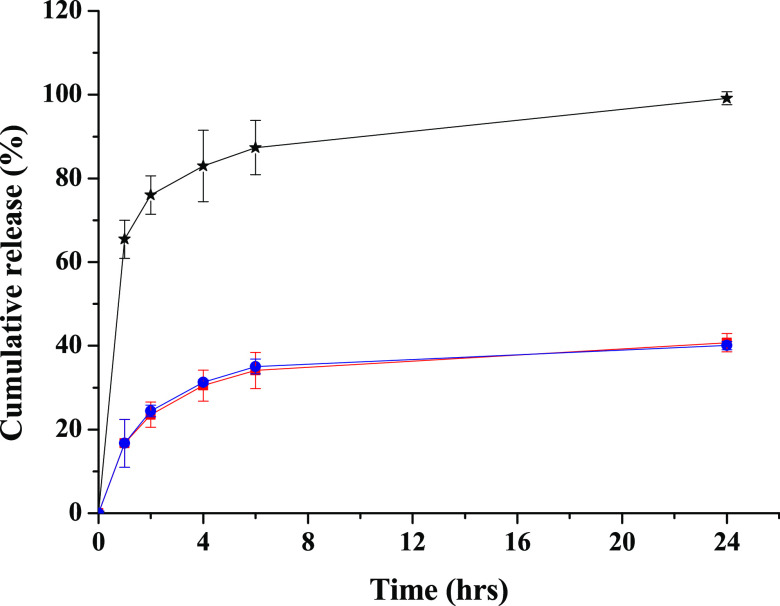
Percentage of Roflumilast released by LPHFNPs (red), Man-LPHFNPs (blue), and diffusion profile (black). Roflumilast release profile from LPHFNPs was realized using the dialysis method, under sink conditions. Each sample was dispersed in 1 mL of PBS at pH 7.4, placed in a dialysis tubing (MWCO 12–14 kDa), immersed into PBS (pH 7.4)/ethanol (80/20, v/v), and then incubated at 37 °C under continuous stirring. At fixed time intervals (1, 2, 4, 6, and 24 h), an aliquot of the external medium was withdrawn, replaced with an equal volume of fresh medium, and freeze-dried, and Roflumilast was quantified by HPLC analysis as reported in the experimental part.

To know the ability of the produced LPHFNPs to retain the entrapped drug under sink conditions and to ensure a sustained release, the Roflumilast release profile from LPHFNPs was evaluated using the dialysis method, and the obtained data are reported in Figure 4. The figure also shows the diffusion profile of the free drug through the membrane, under the same experimental conditions used to study the drug release from hybrid systems.

As shown, there are no differences in kinetics release between the two hybrid systems, with the drug being slowly released in the aqueous medium, without showing burst effect, and the released amount of Roflumilast was about 40 wt % of the total amount after 24 h of incubation.

To obtain preliminary information on the spatial arrangement and the establishment of possible interactions between the components (phospholipids, polymer, and drug) constituting the LPHFNPs, a differential scanning calorimetry (DSC) analysis was performed.^33−35^

**Figure 5:**
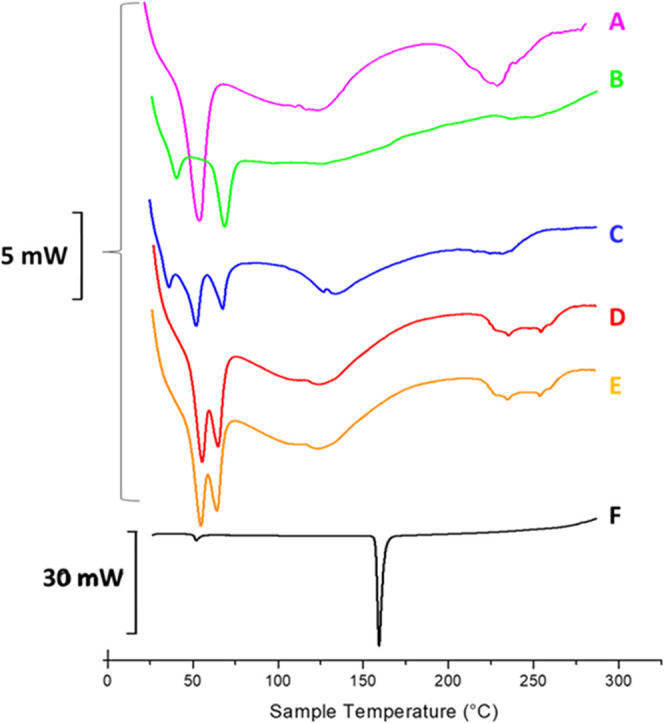
DSC thermogram of (A) FNPs, (B) DPPC/DSPE-PEG_2000_-mannose liposomes, (C) physical mixture of FNPs and liposomes, (D) Man-LPHFNPs, (E) Man-LPHFNPs@Roflumilast, and (F) Roflumilast in the crystalline form. Each sample (5–10 mg) was sealed in an aluminum pan and submitted to heating and cooling cycles in the temperature range of 20–300 °C at a scanning rate of 5 °C/min for heating and a scanning rate of 10 °C/min for cooling.

Specifically, DSC analysis was performed on empty and Roflumilast-loaded Man-LPHFNPs, the starting FNPs, the lipid vesicles, the drug alone, and the physical mixture between the FNPs and the phospholipid-based vesicles. DSC diagrams and transition temperatures of the main peaks are reported in Figure 5 and Table 6, respectively.

**Table 6:** Transition Temperature Values of Analyzed Samples

sample	*T*_M_/°C
FNPs	peak 1, 54.73
	peak 2, 240.0
liposomes	peak 1, 40.43
	peak 2, 68.75
physical mixture	peak 1, 38.19
	peak 2, 52.30
	peak 3, 67.81
Man-LPHFNPs	peak 1, 55.59
	peak 2, 65.03
Man-LPHFNPs@Roflumilast	peak 1, 55.59
	peak 2, 65.03
Roflumilast	159.8

As can be seen in Figure 5, the thermogram of the FNPs (A) shows two endothermic peaks at 54.73 and 240 °C, attributed to the melting and the degradation of the PHEA-g-RhB-g-Succ-PCL copolymer, respectively. The thermogram of the liposomal vesicles (B) shows two peaks, at 40.43 and 68.75 °C, attributed to the sol–gel phase transitions of DSPE-PEG_2000_-mannose and DPPC, respectively. In the case of the physical mixture between FNPs and phospholipid vesicles (C), the thermogram shows three peaks, which overlap perfectly with those present in the thermograms of the FNPs and vesicles, recorded individually. The profiles of both Man-LPHFNPs and Man-LPHFNPs@Roflumilast (D and E) also show endothermic peaks that overlap with those present in the thermograms of the FNPs and liposomes recorded separately, suggesting that the formation of hybrid systems occurred successfully. In addition, the peak relative to the phase transition of DPPC is slightly shifted moving from the physical mixture to the LPHFNPs (67.81 vs 65.03 °C), suggesting that the incorporation into the hybrid systems leads to the occurrence of interactions that affect the sol–gel transition temperature of the latter phospholipid. In the case of DSPE-PEG-Mann, its phase transition peak (40.43 °C) disappears in the thermogram of hybrid systems, probably because of a low relative amount present in the sample.

### Biological Characterization

A major concern relevant to any application of a drug carrier system is its safety. For this reason, the toxicity of all obtained hybrid systems was investigated on the cell lines that are present in the pulmonary compartment, such as 16-human bronchial epithelial (16-HBE) cells and a macrophagic cell line (RAW 264.7). The latter was chosen to evaluate the targeting effect of mannose on the macrophagic cells, which, unlike 16-HBE cells, expose CD-206 receptors on the surface, overexpressed in AMs of COPD patients.^11^

**Figure 6:**
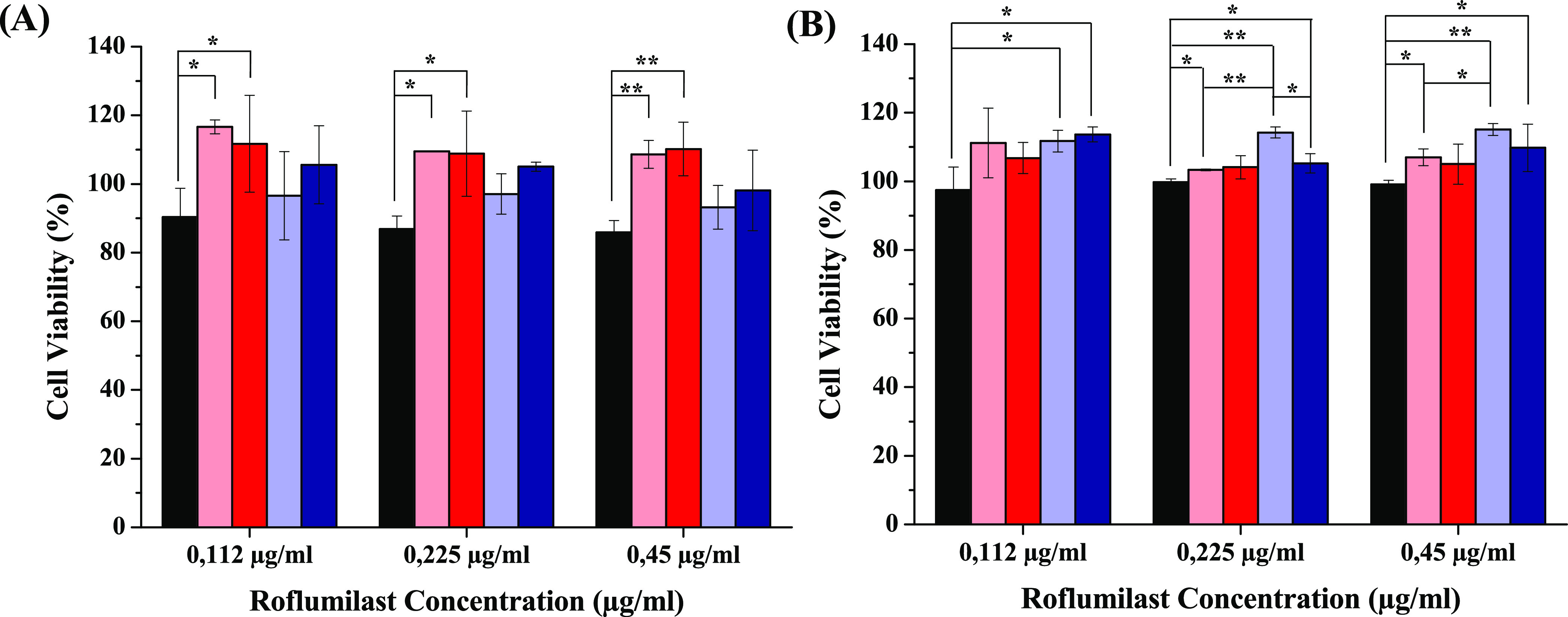
Cell viability assay after 24 h for (A) RAW 264.7 and (B) 16-HBE cells treated with free Roflumilast (black), LPHFNPs (pink), LPHFNPs@Roflumilast (red), Man-LPHFNPs (light blue), and Man-LPHFNPs@Roflumilast (blue). (*P < 0.05; **P < 0.001).

In particular, both cells were incubated for 24 h with different concentrations of Man-LPHFNPs@Roflumilast, LPHFNPs@Roflumilast, and the free drug. Moreover, the effect of empty LPHFNPs on cell viability was also evaluated at concentrations corresponding to those used for the drug-loaded systems. Results are shown in Figure 6A,B.

As can be seen, after 24 h of incubation, all samples showed negligible cytotoxicity at all tested concentrations, with the cell viability of both cell lines being higher than 80% compared to the control experiment. Moreover, RAW 264.7 cells treated with Man-LPHFNPs and Man-LPHFNPs@Roflumilast showed a comparable cytotoxicity effect to each other and the free drug. This effect could be due to the increased internalization of targeted systems thanks to the presence of mannose. On the other hand, the cytotoxicity on 16-HBE of mannosylated systems is significantly lower than that obtained with the free drug, supporting the targeting effect of mannose.

Thanks to the fluorescence properties of our hybrid systems given by RhB, it was evaluated, by fluorescence imaging, whether the uptake of Man-LPHFNPs by RAW 264.7 cells is effectively increased by the contributor of the mannose. Meanwhile, the uptake study was also carried out on 16-HBE cells to confirm the targeting effect of mannose on the Man-LPHFNP systems. In addition, the same uptake experiment was carried out by incubating both cell lines with LPHFNPs as negative controls. In particular, RAW 264.7 and 16-HBE cells were treated with Man-LPHFNPs and LPHFNPs for 4 and 24 h at 37 °C.

**Figure 7:**
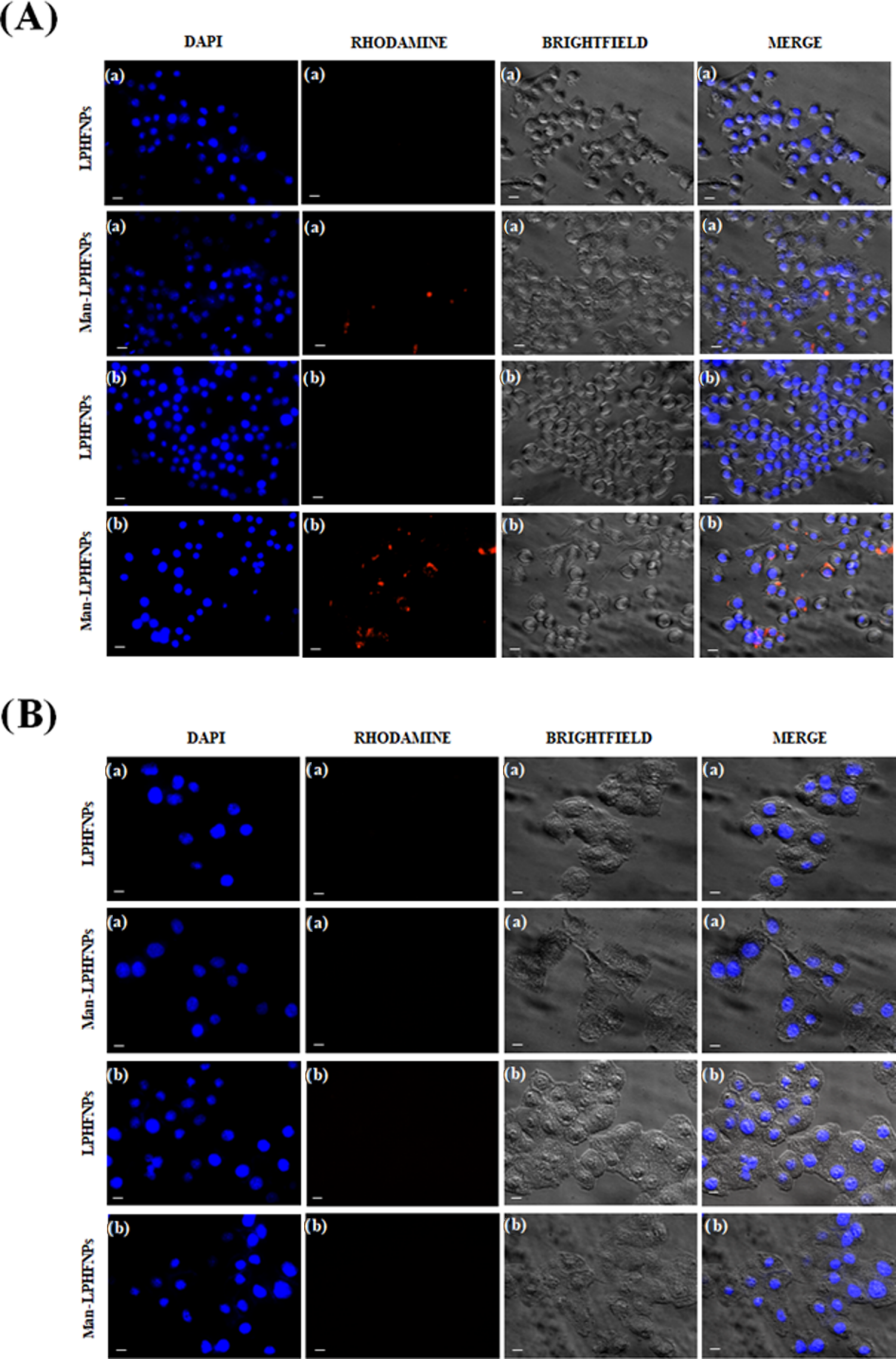
Fluorescence microscopy images of (A) RAW 264.7 cells and (B) 16-HBE cells treated after (a) 4 h and (b) 24 h of incubation. The bar represents 10 μm.

Figure 7A shows Man-LPHFNPs inside RAW 264.7 cells already 4 h after incubation; based on the observed fluorescent intensities, the maximum uptake was noted within 24 h, showing a time-dependent uptake. Despite the phagocytic nature of RAW 264.7, no staining is observed when they are incubated with untargeted systems for 4 and 24 h.

Moreover, the receptor-mediated interactions of Man-LPHFNPs toward RAW 264.7 cells were demonstrated using a competitive inhibition assay by incubating an excess (10 mM) of free mannose with Man-LPHFNPs. In particular, the uptake of Man-LPHFNPs was found to be strongly decreased in the presence of free mannose, confirming the contributory ability and specificity of the CD-206 to internalization (data not shown).

Concerning the uptake assay on 16-HBE, as can be seen in Figure 7B, at the same incubation times, no uptake of either system was found inside these cells.

These results successfully confirm that the preparation of mannosylated formulation represents an excellent strategy for targeting drugs specifically toward alveolar macrophages, according to results obtained by other authors.^24,36−38^

### Preparation and Characterization of NiM Formulations

Nanoparticle powders are not suitable for direct inhalation due to their dimensions, which are not appropriate for bronchial deposition.^13^ One of the most promising approaches to turning nanoparticles into inhalable dry powders is the nano-into-micro strategy (NiM), where nanoparticles are entrapped into water-soluble microparticles. Ideally, when deposited on lung fluids (e.g., mucus), NiM should rapidly dissolve and release embedded colloidal particles, which could efficiently diffuse along the respiratory membranes reaching the epithelial surface.^13^

In our work, NiM particles were obtained by spray-drying an aqueous dispersion of Man-LPHFNPs@Roflumilast containing poly(vinyl alcohol) (PVA) and l-leucine (Leu) (details in Supporting Materials). The yield of obtained particles was about 40 wt % based on the theoretical amount.

PVA and Leu, largely used as pharmaceutical excipients, were chosen because of their ability to minimize particle aggregation during powder aerosolization and to improve the aqueous redispersibility of powders after the spray-drying process.^12,14^

**Figure 8:**
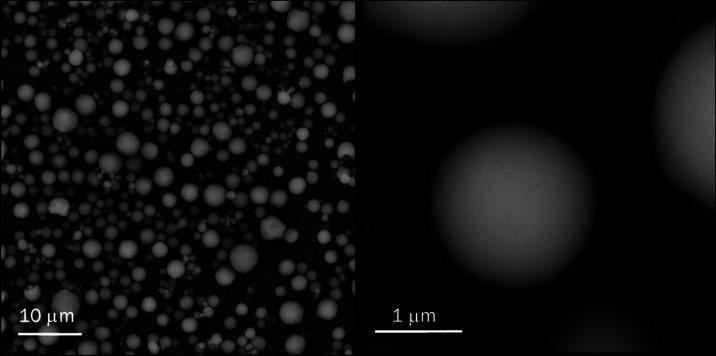
SEM images of NiM particles (sn 5000×, dx 66000×).

After production, the particle morphology and diameter were investigated by scanning electron microscopy (SEM) (analyzed as powder). Representative images are reported in Figure 8.

We obtained NiM particles with a spherical shape and a mean diameter of 1.87 ± 1.03 μm, calculated with the help of the software ImageJ, and therefore morphologically and dimensionally suitable for optimal lung deposition.

Redispersibility of Man-LPHFNPs@Roflumilast was evaluated with DLS measurement by dissolving a certain amount of NiM in water. After microparticle dissolution, Man-LPHFNPs@Roflumilast showed both size and ζ potential similar to that obtained after the freeze-drying process, which were 120.23 ± 2.4 nm and −17.2 ± 4.5 mV, respectively, showing that the spray-drying process does not alter particle technological properties.

## Conclusions

Here, an innovative pulmonary formulation, based on lipid–polymer hybrid nanoparticles (LPHFNPs), was produced for Roflumilast delivery to alveolar macrophages, potentially useful for the management of COPD. Roflumilast is a unique selective PDE4 inhibitor, with an anti-inflammatory effect, approved for the oral treatment of COPD.^4^

To achieve this, first, an amphiphilic graft copolymer was synthesized starting from α,β-poly(*N*-2-hydroxyethyl)-d,l-aspartamide (PHEA), and polymeric fluorescent nanoparticles (FNPs), containing Roflumilast, were produced by nanoprecipitation.

Considering the advantages of using hybrid nanosystems in the drug delivery field, the Roflumilast-loaded FNPs were properly combined with liposomal vesicles prepared starting from a mixture of DPPC and DSPE-PEG_2000_-mannose phospholipids, to obtain hybrid nanoparticles (Man-LPHFNPs@Roflumilast) targeted to receptors for lectins, overexpressed on the macrophage membrane of COPD patients.

Man-LPHFNPs@Roflumilast showed sizes in the nanometer range (∼100–150 nm), exhibiting high size uniformity and a negative ζ potential. Due to their composition, these can be freeze-dried without the use of any cryoprotectants as they redisperse after drying without giving aggregates. Moreover, once redispersed in a physiological medium, they were able to release the entrapped drug in a controlled manner, without burst effect.

The characterization of the obtained powder by a colorimetric assay for the quantification of the lipid content, XPS surface, STEM, and DSC analysis demonstrates the incorporation of the FNPs into the lipid vesicles and the effective formation of the Man-LPHFNPs@Roflumilast with a core–shell structure. In addition, the determination of surface pegylation density confirmed the presence of PEG chains on the surface of Man-LPHFNPs with a brushlike conformation, necessary to impart mucopenetrating properties to the nanosystems and to assure exposition of the targeting ligand. Considering the size, ζ potential, surface pegylation density, and conformation of the PEG chains on the surface of Man-LPHFNPs, we can assume that, once administered, they have the ability to diffuse through the mucus layer. This theory is supported by the results obtained from the turbidimetric and rheological analyses, which excluded significant mucoadhesive interaction with artificial mucus components.

Cell viability studies, carried out on 16-HBE and RAW 264.7 cells, showed low cytotoxicity for all hybrid samples (higher than 80%). In particular, RAW 264.7 cells treated with both empty or drug-loaded Man-LPHFNPs showed a cytotoxic effect comparable to that of the drug, probably due to internalization mediated by the presence of mannose on the surface. This effect has been confirmed by uptake studies. In particular, it was demonstrated that Man-LPHFNPs are endocytosed by RAW 264.7 cells, and the uptake was annulated in the presence of free Mannose. Moreover, no uptake of untargeted particles by the same cells was observed, demonstrating the key role of mannose and the CD-206 receptor in the internalization process.

Moreover, to overcome the aerodynamic limitations of the nanocarrier for inhalation, a pulmonary drug-delivery system composed of mucus-penetrating Man-LPHFNPs@Roflumilast and poly(vinyl alcohol) (PVA) and l-leucine (Leu) was obtained using the NiM strategy and realized by spray-drying. Spherical NiM particles with suitable dimensions for an optimal lung deposition were produced.

Considering these encouraging results, the obtained formulation could represent a good candidate as a carrier for the pulmonary delivery of Roflumilast for the treatment of COPD.

## Supporting Information Available

^1^H-NMR spectra, SEC chromatograms, FT-IR spectra, distribution curves of mean size (intensity %), and zeta potential (PDF)
