# Supplementary material for: Inhalable Formulation Based on Lipid–Polymer Hybrid Nanoparticles for the Macrophage Targeted Delivery of Roflumilast
Source: Biomacromolecules. 2022 Jul 28;23(8):3439–51. doi: 10.1021/acs.biomac.2c00576 (PMC9364311; doi:10.1021/acs.biomac.2c00576)
Supplement: Supplementary file 1 — bm2c00576_si_001.pdf [file bm2c00576_si_001.pdf]

Supporting Information

Inhalable formulation based on lipid-polymer hybrid nanoparticles for the macrophage targeted delivery of Roflumilast

*Emanuela F. Craparo[1,2], Marta Cabibbo[1], Cinzia Scialabba[1], Gaetano Giammona[1,2], Gennara Cavallaro[1,2,3]**

[1]Lab of Biocompatible Polymers, Department of Biological, Chemical and Pharmaceutical Sciences and Technologies (STEBICEF), University of Palermo, Via Archirafi 32, Palermo, 90123, Italy

[2]Consorzio Interuniversitario Nazionale per la Scienza e Tecnologia dei Materiali (INSTM) of Palermo.

[3]Advanced Technology and Network Center (ATeN Center), Università di Palermo, Palermo 90133, Italy;

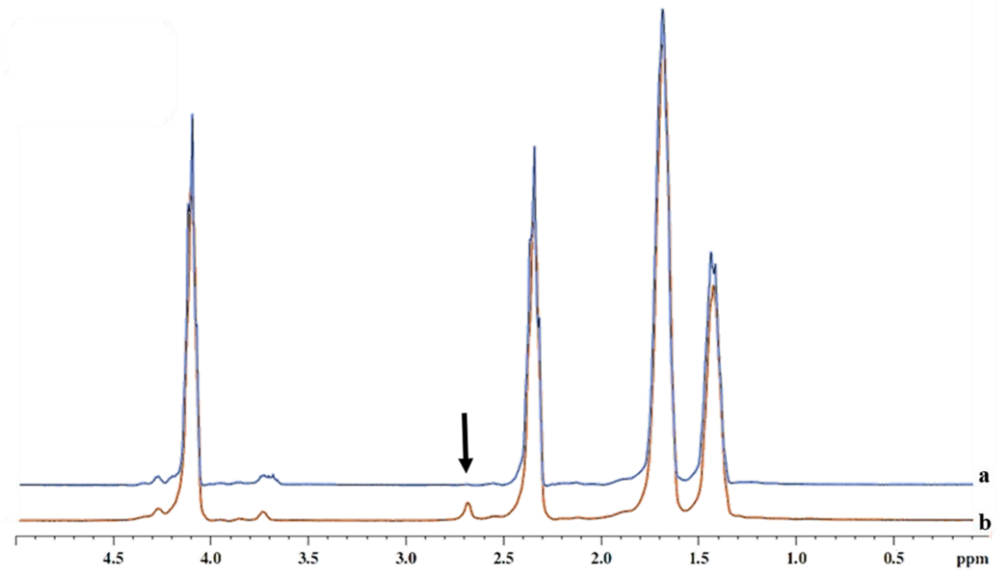

Figure S1. ^{1}H NMR spectra of PCL and PCL-Succ in $CDCl_3$.

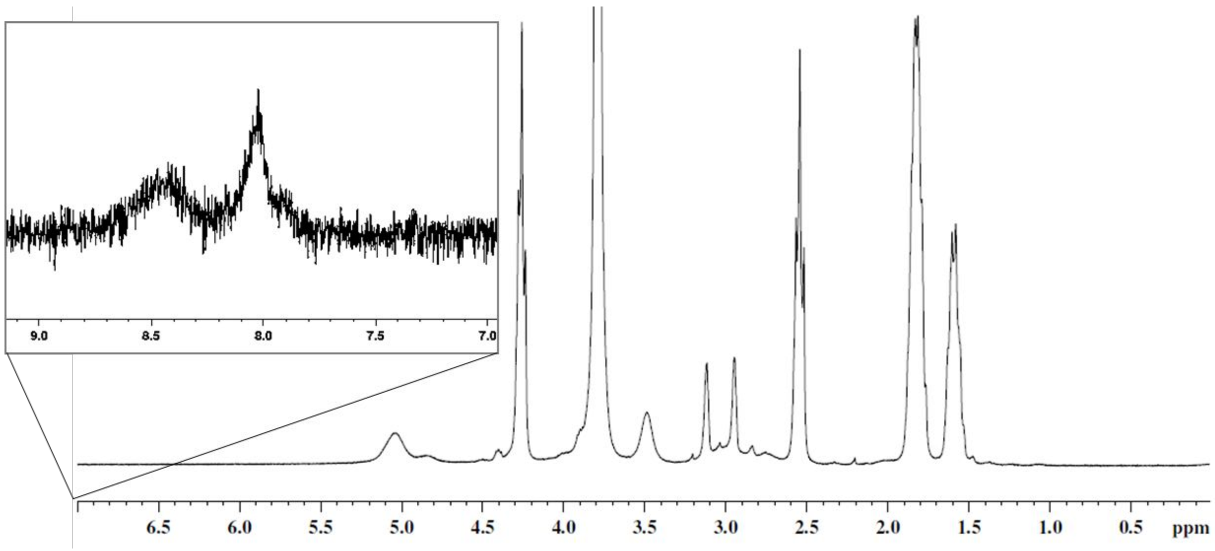

Figure S2. ^{1}H-NMR spectrum of PHEA-g-RhB-g-SUCC-PCL copolymers in DMF-d7.

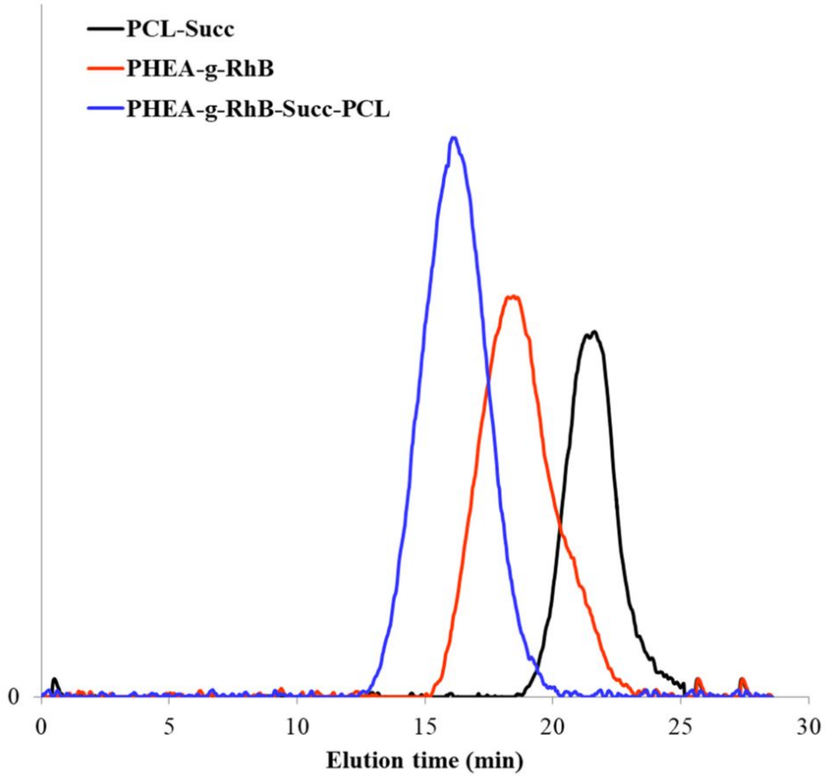

Figure S3. Size Exclusion Chromatography (SEC) chromatograms for PCL-Succ (black line), PHEA-RhB (red line) and PHEA-g-RhB-g-Succ-PCL graft copolymer (blue line) in DMF + LiBr 0.01M.

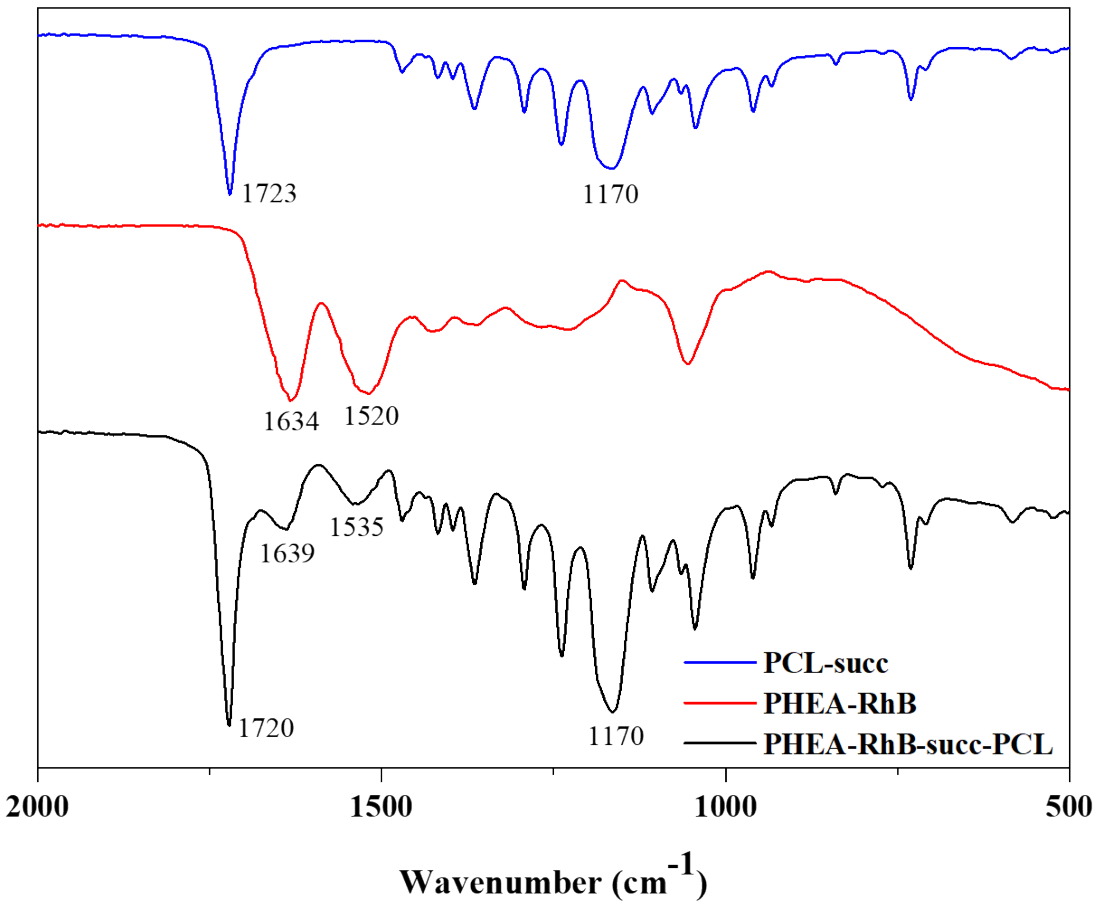

Figure S4. FT-IR spectra of PCL-Succ (blue line), PHEA-g-RhB (red line), and PHEA-g-RhB-g-Succ-PCL graft copolymer (black line). Each freeze-dried copolymer was analysed and spectra were registered by a Bruker ALPHA FT-IR spectrometer (Bruker, Milan, Italy).

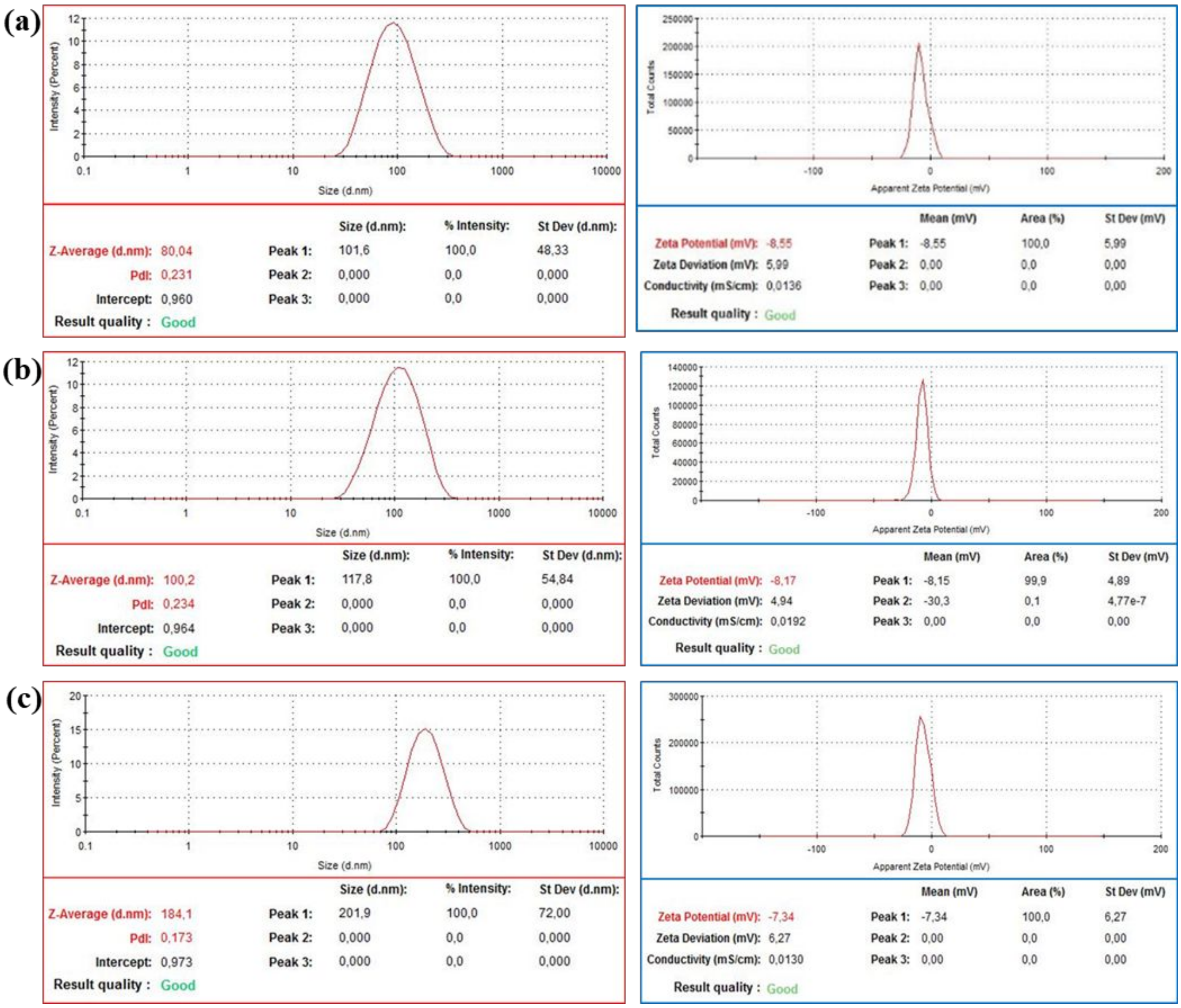

Figure S5. Distribution curves of mean size (intensity %) (left) and zeta potential (right) of sample LPHFNPs before freeze-drying (a), and after freeze-drying and redispersion in bidistilled water (b) or in a 10 mM NaCl aqueous solution (c). Both analyses were obtained by using a Malvern Zetasizer NanoZS (Malvern Instruments).

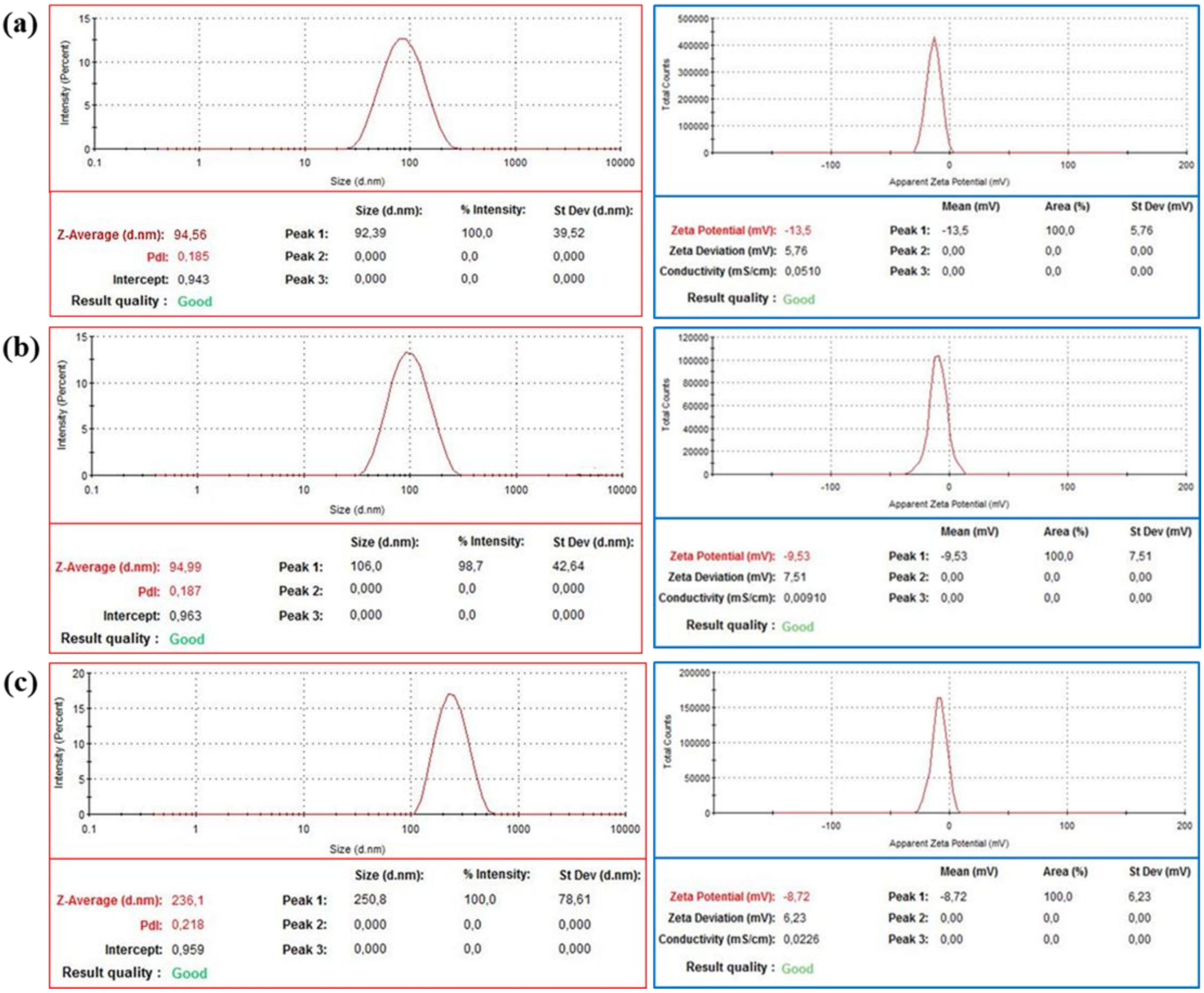

Figure S6. Distribution curves of mean size (intensity %) and zeta potential of sample LPHFNPs@Roflumilast before freeze-drying (a), and after freeze-drying and redispersion in bidistilled water (b) or in a 10 mM NaCl aqueous solution (c).

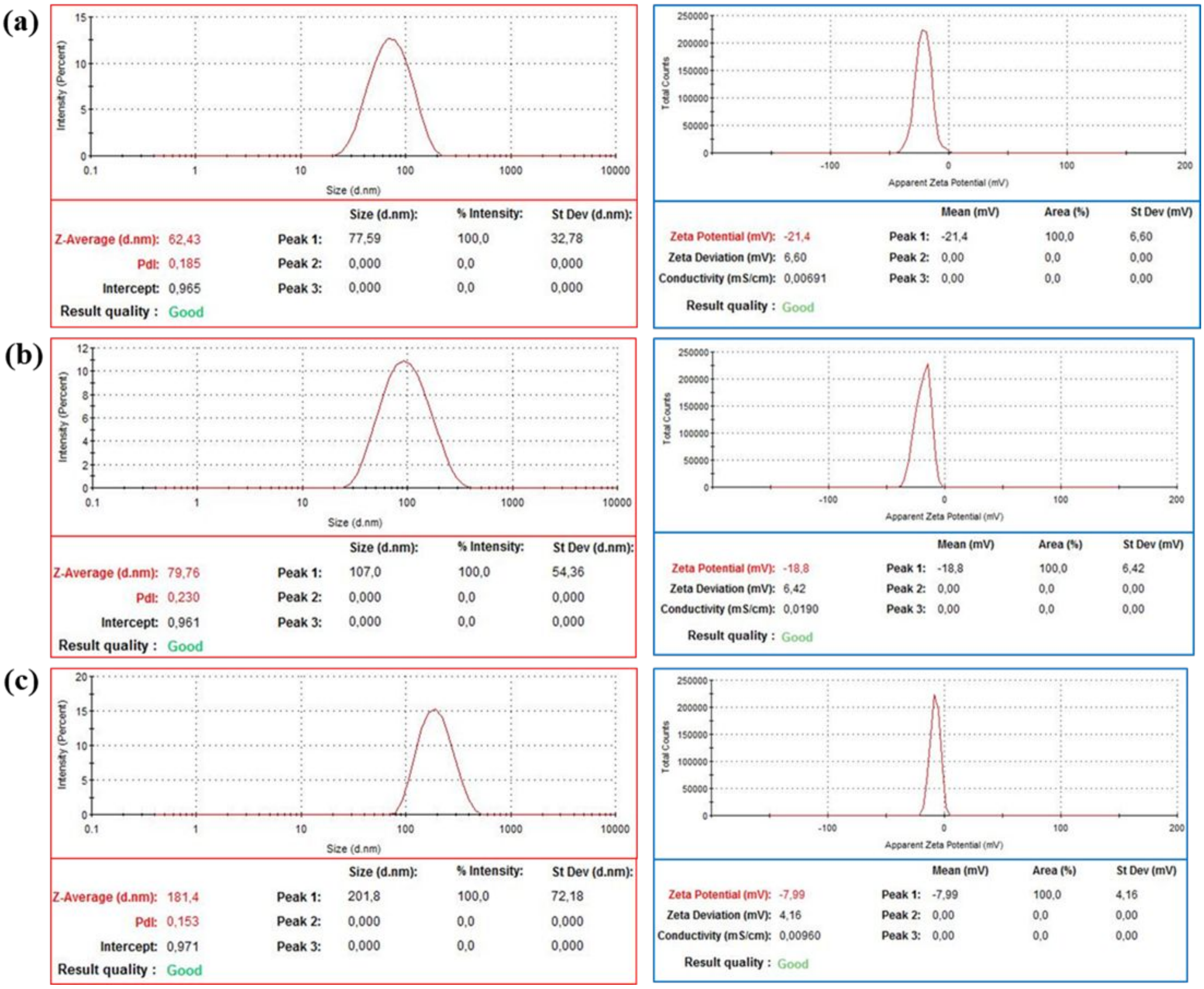

Figure S7. Distribution curves of mean size (intensity %) and zeta potential of sample Man-LPHFNPs before freeze-drying (a), and after freeze-drying and redispersion in bidistilled water (b) or in a 10 mM NaCl aqueous solution (c).

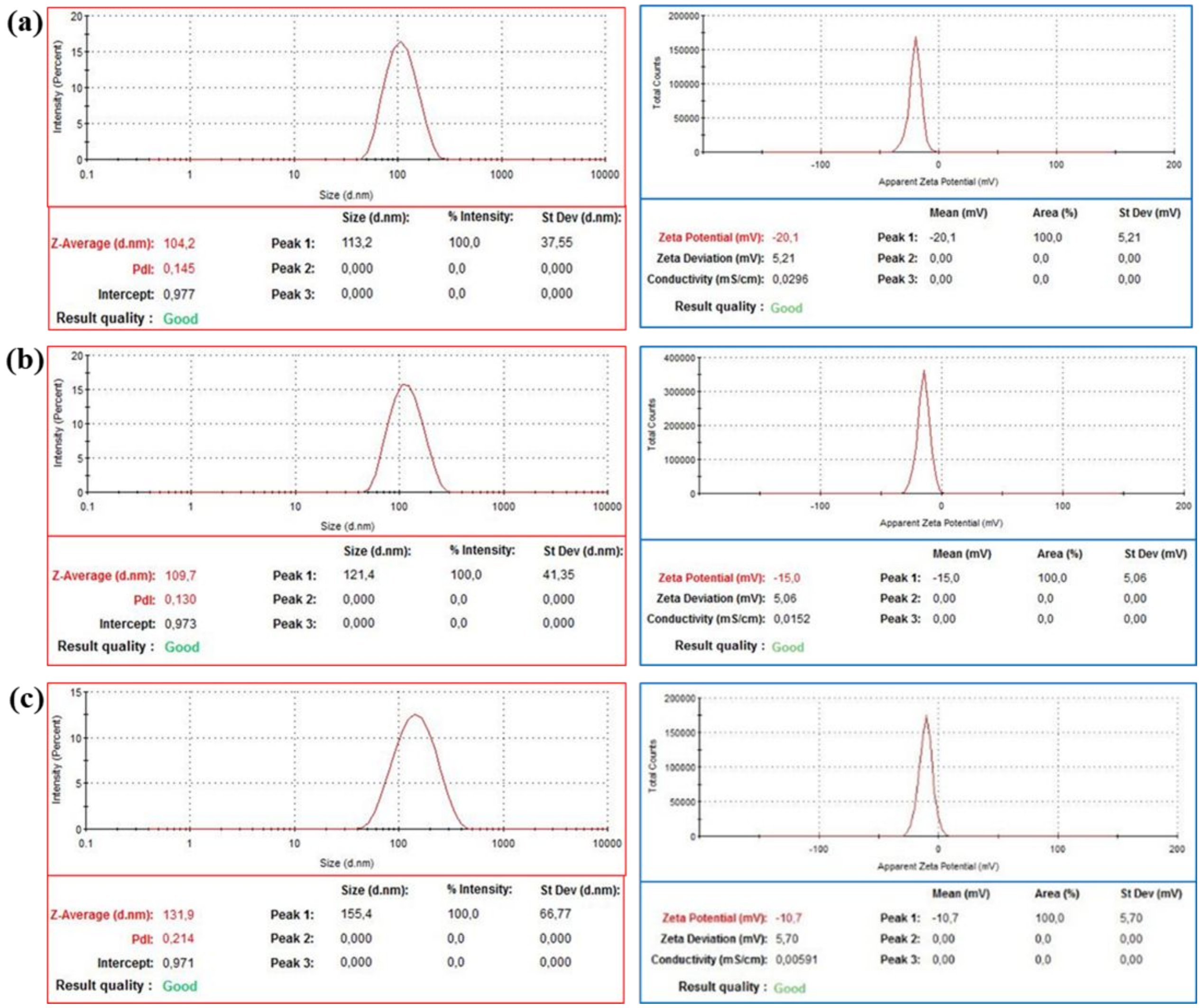

Figure S8. Distribution curves of mean size (intensity %) and zeta potential of sample Man-LPHFNPs@Roflumilast freeze-drying (a), and after freeze-drying and redispersion in bidistilled water (b) or in a 10 mM NaCl aqueous solution (c).
